# Farnesyltransferase inhibitor LNK-754 attenuates axonal dystrophy and reduces amyloid pathology in mice

**DOI:** 10.1186/s13024-022-00561-9

**Published:** 2022-08-20

**Authors:** Leah K. Cuddy, Alia O. Alia, Miranda A. Salvo, Sidhanth Chandra, Tom N. Grammatopoulos, Craig J. Justman, Peter T. Lansbury, Joseph R. Mazzulli, Robert Vassar

**Affiliations:** 1 The Ken and Ruth Davee Department of Neurology, Northwestern University Feinberg School of Medicine, Chicago, IL 60611 USA; 2 BioEnergetics, Boston, MA 02115 USA; 3 Bial Biotech, Cambridge, MA 02139 USA; 4 Department of Neurology, Harvard Medical School, Cambridge, MA 02139 USA; 5 Mesulam Center for Cognitive Neurology and Alzheimer’s Disease, Northwestern University Feinberg School of Medicine, Chicago, IL 60611 USA

**Keywords:** Alzheimer’s disease, Dystrophic neurites, Farnesyltransferase inhibitors

## Abstract

**Background:**

Amyloid plaque deposition and axonal degeneration are early events in AD pathogenesis. Aβ disrupts microtubules in presynaptic dystrophic neurites, resulting in the accumulation of impaired endolysosomal and autophagic organelles transporting β-site amyloid precursor protein cleaving enzyme (BACE1). Consequently, dystrophic neurites generate Aβ42 and significantly contribute to plaque deposition. Farnesyltransferase inhibitors (FTIs) have recently been investigated for repositioning toward the treatment of neurodegenerative disorders and block the action of farnesyltransferase (FTase) to catalyze farnesylation, a post-translational modification that regulates proteins involved in lysosome function and microtubule stability. In postmortem AD brains, FTase and its downstream signaling are upregulated. However, the impact of FTIs on amyloid pathology and dystrophic neurites is unknown.

**Methods:**

We tested the effects of the FTIs LNK-754 and lonafarnib in the 5XFAD mouse model of amyloid pathology.

**Results:**

In 2-month-old 5XFAD mice treated chronically for 3 months, LNK-754 reduced amyloid plaque burden, tau hyperphosphorylation, and attenuated the accumulation of BACE1 and LAMP1 in dystrophic neurites. In 5-month-old 5XFAD mice treated acutely for 3 weeks, LNK-754 reduced dystrophic neurite size and LysoTracker-Green accumulation in the absence of effects on Aβ deposits. Acute treatment with LNK-754 improved memory and learning deficits in hAPP/PS1 amyloid mice. In contrast to LNK-754, lonafarnib treatment was less effective at reducing plaques, tau hyperphosphorylation and dystrophic neurites, which could have resulted from reduced potency against FTase compared to LNK-754. We investigated the effects of FTIs on axonal trafficking of endolysosomal organelles and found that lonafarnib and LNK-754 enhanced retrograde axonal transport in primary neurons, indicating FTIs could support the maturation of axonal late endosomes into lysosomes. Furthermore, FTI treatment increased levels of LAMP1 in mouse primary neurons and in the brains of 5XFAD mice, demonstrating that FTIs stimulated the biogenesis of endolysosomal organelles.

**Conclusions:**

We show new data to suggest that LNK-754 promoted the axonal trafficking and function of endolysosomal compartments, which we hypothesize decreased axonal dystrophy, reduced BACE1 accumulation and inhibited amyloid deposition in 5XFAD mice. Our results agree with previous work identifying FTase as a therapeutic target for treating proteinopathies and could have important therapeutic implications in treating AD.

**Supplementary Information:**

The online version contains supplementary material available at 10.1186/s13024-022-00561-9.

## Background

Alzheimer’s disease (AD) is a progressive neurodegenerative disease that is the most common cause of dementia in the aging population and is characterized by significant impairment in learning and memory. At the molecular level, hallmark features of the AD brain are the buildup of amyloid-β (Aβ) plaques and the presence of hyperphosphorylated tau inclusions. In the early, asymptomatic stages of AD, Aβ accumulates in the brain for over two decades or longer prior to the onset of memory impairment [1–3]. Consequently, reducing brain Aβ levels has been the central focus of treatment strategies for AD [4]. Currently, the only disease-modifying therapy conditionally approved by the FDA is an anti-Aβ antibody that clears aggregated forms of Aβ from the brain [5], confirming that targeting Aβ in the brain is a promising approach to AD treatment. However, most clinical trials focused on Aβ have failed due to safety or efficacy concerns, and emphasis has now been placed on targets based on different hypotheses of AD, including tau propagation, inflammation, calcium dyshomeostasis and neurovascular factors [4, 6, 7]. In addition, there is growing interest in the therapeutic potential of activating lysosomal degradative pathways as a method to reduce pathogenic proteins, a feature common across neurodegenerative diseases [8–12].

Extensive dysfunction of endolysosomal and autophagic pathways is well-documented as an early event AD pathogenesis. Many AD susceptibility genetic loci are linked to subcellular endolysosomal transport and lysosome function [13], and enlarged dysfunctional endolysosomal and autophagic organelles accumulate in the AD brain within dystrophic neurites [14, 15]. Dystrophic neurites appear to be initiated upon neuronal injury, and in AD this is proposed to be the destabilization of microtubules by Aβ [16]. This results in focal axonal swellings that disrupt the normal trafficking and degradation pathways of key proteins involved in Aβ production, including BACE1 [16, 17]. As a result, dystrophies generate Aβ42 and significantly contribute to extracellular plaque deposition and axonal dystrophy [18], initiating a feed-forward pathway resulting in synaptic loss, neurodegeneration, and cognitive deficits [16]. Therapeutic strategies that improve the transport and activity of endolysosomal and autophagic organelles could prevent neuritic dystrophy and sustain the degradation of pathogenic proteins, and be a critical mechanism to reducing BACE1 and Aβ levels in AD.

As the search continues for an effective AD treatment, the number of repurposed drugs in clinical trials for AD has grown substantially as clinically evaluated drugs have known safety and efficacy profiles, thus accelerating the drug development process [7, 19]. In recent years, studies have identified FTIs as being a class of drugs with the potential to repurpose towards treating neurodegenerative diseases. FTIs inhibit farnesylation, a post-translational modification that regulates proteins in which farnesyl pyrophosphate is covalently modified to cysteine residues in proteins that contain a CAAX motif [20]. FTIs were initially designed to block oncogenic Ras activity by inhibiting its prenylation, but were discontinued in clinical trials for cancer treatment following the discovery of alternate Ras prenylation pathways [21, 22]. However, many FTase substrates exist [23–25], leading to the evaluation of FTIs for a wide range of disorders, including cardiovascular disease, hepatitis D and progeria [20, 26]. Additionally, FTIs have shown promise towards neurodegenerative diseases characterized by the accumulation of protein aggregates. In rodent and cell culture models of proteinopathies, FTIs induce lysosomal activity and the proteolysis of disease-specific toxic proteins [8, 9, 27, 28].

Previously, we showed that LNK-754, an FTI tested in cancer trials, enhanced lysosomal hydrolase trafficking, and decreased alpha-synuclein (α-synuclein) aggregates in Parkinson’s disease (PD) patient-derived neurons and PD mouse models [8]. Other studies have reported similar results of FTIs towards reducing protein aggregates; treating cell culture models of PD with FTI-277 reduced α-synuclein [28] and lonafarnib, an FDA-approved FTI for the treatment of progeria [29], increased lysosomal activity and reduced tau inclusions in a mouse model of tauopathy [9]. Furthermore, a recent study showed that genetic knockout of neuronal FTase in the APP/PS1 mouse model of AD prevented Aβ generation [30]. The focus of past studies evaluating FTIs for proteinopathies has been the role of FTase substrates in lysosome activity and autophagy. However, FTIs are also known to promote microtubule (MT) stability and synergize with MT-stabilizing drugs to enhance tubulin acetylation, through a Ras independent pathway [31–33]. MT stabilization inhibits AD pathology in APP/PS1 mice [34], and currently, several MT stabilizing agents are being investigated clinically for the treatment of AD [35]. Therefore, substantial evidence suggests that FTIs may benefit diseases characterized by dysfunctional protein homeostasis mechanisms such as AD. Still, the targets and pathways that connect protein farnesylation with decreased protein aggregates in neurodegenerative diseases are not fully understood.

In this study, we assessed whether pharmacological treatment with FTIs can improve Aβ-associated AD pathology. We evaluated the effects of LNK-754 and lonafarnib, two FTIs known to reduce protein aggregates in neurodegenerative disorders, on plaque burden and dystrophic neurites in the 5XFAD mouse model of amyloid pathology. Our results provide insights into the pathways affected by FTase inhibition and strongly support further investigation into the repurposing of FTIs as a therapeutic for AD and other neurodegenerative diseases characterized by protein aggregation buildup.

## Materials and methods

### Animals and drug treatments

All animal work was done in accordance with Northwestern University Institutional Animal Care and Use Committee (IACUC) approval. Mice were fed a standard rodent chow diet and water ad libitum, and housed with a standard 12-hour light/dark cycle. 5XFAD mice on a C57BL6 background (Jackson Laboratories) were maintained by crossing transgene positive hemizygous male C57BL6 mice with transgene negative female C57BL6 mice (Jackson Laboratories). In our chronic treatment protocol, drug treatments were initiated at 2 months old, the age at which plaque deposition is beginning [36, 37]. In the acute treatment protocol, drug treatment was initiated at 5 months of age after which plaque deposition and axonal degeneration is widespread [38, 39].

LNK-754 was synthesized by Link Medicine (Massachusetts USA) and lonafarnib was obtained from Cayman Chemical Company (Ann Harbor, MI). For animal treatments, LNK-745 and lonafarnib were prepared in a heated solution of 0.5% carboxymethylcellulose (Sigma-Aldrich) as a vehicle at a concentration of 0.10 mg/mL until dissolved and the solution was clear. Aliquots for injection were frozen at − 20 °C and then thawed and stored at 4 °C for each week of treatment. Mice were treated following an established protocol known to enhance lysosome activity [8]. In the chronic treatment protocol, 2-month-old 5XFAD mice were injected by intraperitoneal (i.p) injection with either vehicle, LNK-754 or lonafarnib daily using a 29-guage insulin syringe for a 12-week period until mice were 5 months old. In the acute treatment regimen, 5-month-old 5XFAD mice were i.p injected daily for a 3-week period. Upon completion of treatments, mice were anesthetized, perfused, and brains were collected for further analyses (see below sections). For cell culture experiments, LNK-754 and lonafarnib were dissolved in DMSO and added to mouse primary neuron cultures at a concentration of 10 nM for a 48-hour period. The vehicle treatment was an equivalent amount of DMSO. Cells were collected and analyzed as described below.

### Antibodies

The antibodies used for immunofluorescence experiments were rat anti-LAMP1 (#1D4B, DSHB), rabbit anti-Aβ42 (#700254, Invitrogen), rabbit anti-BACE1 (#ab108394, Abcam), rabbit anti-βIII tubulin (#ab18207, Abcam). For immunoblotting, the antibodies used were rabbit anti-actin (#926–42,210, LI-COR), rabbit anti-p44/42 MAPK (pErk1/2) (#CS9101, Cell Signaling Technology), rabbit anti-44/42 (Erk1/2) (#CS137F5, Cell Signaling Technology), rabbit anti-LAMP1 (#CS3234, Cell Signaling Technology), mouse anti-BACE (3D5; Zhao et al., 2007), mouse anti-APP (6E10) (#803001, BioLegend), rabbit anti-p-tau (ser404) (#CS20194, Cell Signaling Technology), mouse anti-Tau1 (#MAB3420, Millipore Sigma), prelamin-A, (#MABPT858, Millipore Sigma) and mouse anti-HDJ-2 (#sc-59,554, Santa Cruz). Anti-rabbit or -mouse secondary antibodies from Vector Laboratories were used for immunoblots.

### Behavioral testing

#### Animals and drug treatment

Behavioral analyses were performed by reMYND, Inc. using the hAPP/PS1 mouse model with neuron-specific expression of a clinical mutant of the human amyloid precursor protein together with a clinical mutant of human presenilin 1 [40]. This double transgenic mouse displays spontaneous, progressive accumulation of Aβ in the brain, eventually resulting in amyloid plaques within subiculum, hippocampus and cortex at 6 months of age. 48 female hAPP/PS1 mice in FVB x C57Bl/6 J background of 6 months of age were used for this study, in combination with 12 age-matched female wild type controls in FVB x C57Bl/6 J background. Administration of 0.9 mg/kg/day of LNK-754 was performed per oral gavage, once a day, during 12 consecutive days. The sacrifice of the animals was performed 2 hours after final dosing.

All mice were genotyped by polymerase chain reaction (PCR) at the age of 3 weeks and received a unique identity number, once the PCR results were known. All mice were double checked by a second PCR before the onset of the study. All mice were blind randomized and age-matched. Mice were given a random number by computer and allocated randomly to a treatment. The animals were re-caged by treatment group 15 days before the onset of the study to allow them to familiarize to the new cage context. Mice had free access to pre-filtered and sterile water and standard mouse chow (Sniff® Ms-H, Sniff Spezialdiäten GmbH, Soest, Germany). The food was stored under dry and cool conditions in a well-ventilated storage room. The amount of water and food was checked daily, supplied when necessary and standard refreshed once a week. Mice were initially housed under a reversed day-night rhythm (14 hours light/10 hours darkness) starting at 7 p.m. in standard metal cages type RVS T2 (area of 540 cm^2^). The cages are equipped with solid floors and layer of bedding litter. The number of mice per cage was limited in accordance with local legislation on animal welfare. 15 days before the start of treatment, mice were re-caged in individual ventilated macrolon T2 cages and transported to the laboratory for acclimation to the laboratory environment in preparation for treatment. In the laboratory, the day-night rhythm was changed to 12 hours light/ 12 hours darkness, starting at 7 p.m.

#### Morris water maze

The Morris Water Maze was performed on days 8–12 of treatment, and the animals were sacrificed directly after the probe test. The pool (a white, circular vessel 1 m in diameter) contained water at 20 °C with titanium-dioxide as an odorless, nontoxic additive to hide the escape platform (1 cm beneath the water level). Swimming of each mouse was videotaped and analyzed (Ethovision, Noldus information Technology, Wageningen, the Netherlands). Prior to training, each mouse was placed on top of the platform for 15 seconds. For place navigation tests, mice were trained to locate the hidden platform in seven blocks of three trials over four consecutive days. Each trial consists of a forced swim test of maximum 120 seconds, followed by 10 minutes of rest. The time each mouse needed to locate the platform was measured. The seven consecutive blocks result in a learning curve. 24 hours after the last training, each animal had a probe trial with the platform removed. Mice were allowed to search for 60 seconds, and quadrant search time and crossings of the original platform position was measured. During the final probe test, mice were allowed to search the previous location of the platform during 60 seconds after the platform was removed.

### Primary neuron culture

Forebrains were dissected in Hanks’ Balanced Salt solution (HBSS) (Thermo Fisher #14185–052) from pregnant wild-type C57BL6 mice (Jackson) on E15.5 to obtain primary neurons. Dissected forebrains were digested in 2.5% trypsin at 37 °C for 20 minutes. A fire-polished Pasteur pipette was used to dissociate the neurons in HBSS. The neurons were then plated on a poly-L-lysine-coated six-well plate, 2 × 10^6^ neurons per well in neurobasal medium supplemented with 2% B-27, 500 μM glutamine, 10% horse serum, and 2.5 μM glutamate (all from Thermo Fisher). The media was changed 2 hours later to Neurobasal with 2% B-27, 500 μM glutamine, and 2.5 μM glutamate. 24 hours later, the media was changed to Neurobasal plus 2% B-27 and 500 μM glutamine. Half media changes were made every 2–3 days. Primary neurons were cultured for 7 days and treated with vehicle (DMSO) or 10 nM concentrations of LNK-754, or lonafarnib for 48 hours. Neurons were then fixed or live imaged as described below. For fixation, neurons were incubated for 20 minutes in 4% paraformaldehyde, 0.12 M sucrose in 1XPBS, permeabilized in 0.5% Triton X-100, and incubated with primary antibodies overnight followed by donkey anti-mouse Alexafluor-conjugated secondary antibodies (Molecular Probes, 1:1000) and co-stained with 300 nM DAPI. Coverslips were mounted using Prolong Gold (Molecular Probes), and images acquired on a Nikon A1 confocal microscope with a 60X objective (NA 1.4) and NIS Elements software.

### Live imaging of neurons

For live-imaging experiments, neurons were prepared as above and plated in 35 mm glass bottom dishes (MatTek # P35G-1.5-14C) for 7 days in culture. 10 nM LNK-754 or lonafarnib were then added to conditioned media, control cultures were treated with DMSO as a vehicle. After 48 hours of treatment, media was replaced with conditioned media from untreated parallel plates of neurons. 25 nM of LysoTracker-Green DND-26 (#L7526, Invitrogen) was added to the conditioned media and neurons were incubated for 30 minutes at 37 °C. Time-lapse imaging of neurons was performed using a Ti2 widefield microscope. Imaging began immediately at 30 minutes and continued for no longer than 30 minutes. 1-s exposures were taken every minute, and 20–30 separate areas per dish were imaged within 30 mins. The distance and velocity quantifications of LysoTracker particles and kymograph analyses were performed using NIS Elements software and described in detail below. In separate experiments, 1 μM Lysosensor-Green DND-189 (#L7535, Invitrogen) was added to conditioned media and captured images of live neurons were taken for no longer than 15 minutes using a Nikon A1 confocal microscope with a 60X objective (NA 1.4) and NIS Elements software.

### Live imaging of brain tissues

5-month-old 5XFAD mice were i.p injected daily for a 3-week period and upon treatment completion, mice were anesthetized, and perfused with a solution containing 1 M KCl, 1 M MgCl_2_, 0.1 mM kynurenate, 0.01 mM DL-2-Amino-5-phosphonopentanoic acid, 5 mM glutathione, 25 mM glucose, 125 mM NaCl, 1.4 mM NaH_2_PO_4_ and 25 mM NaHCO_3_. Brains were then rapidly dissected in ice-cold oxygenated ACSF containing 2.5 mM KCl, 85 mM NaCl, 1.25 mM NaH_2_PO_4_, 25 mM NaHCO_3_, 0.5 mM CaCl_2_, 4 mM 4 MgCl_2_, 75 mM sucrose, 25 mM glucose, 50uM C_6_H_7_NaO_6_, 0.1 mM kynurenate, 0.01 mM DL-2-Amino-5-phosphonopentanoic acid and 5 mM glutathione. 300 μm coronal slices were obtained using a Compresstome VF-200 vibrating microtome (Precisionary Instruments) and allowed to rest for 30 minutes for recovery at 32 °C in oxygenated ACSF solution containing the following 2.5 mM KCl, 85 mM NaCl, 1.25 mM NaH_2_PO_4_, 25 mM NaHCO_3_, 0.5 mM CaCl_2_, 4 mM 4 MgCl_2_, 75 mM sucrose, 25 mM glucose, 50uM C_6_H_7_NaO_6_, 0.1 mM kynurenate, 0.01 mM DL-2-Amino-5-phosphonopentanoic acid and 5 mM glutathione. The slices were then transferred to 35 mm glass bottom dishes (MatTek # P35G-1.5-14C) containing oxygenated ACSF with 25 nM LysoTracker-Green and a 1:10,000 dilution of 1 mg/mL Thiazine Red (#S570435, Sigma Aldrich) that was otherwise identical to the ACSF used during the recovery period. Slice viability was confirmed using time-lapse imaging of tissues. Slices were incubated with LysoTracker-Green and Thiazine Red for 15 minutes at room temperature and then cortical brain regions were imaged immediately in the same solution at 32 °C on a Nikon A1 confocal microscope with a 60X objective (NA 1.4) and NIS Elements software for no longer than 30 minutes.

### Brain extraction and immunoblotting

Mice were anesthetized by intraperitoneal injection of xylazine (15 mg/kg) and ketamine (100 mg/kg), perfused with phosphate-buffered saline (1XPBS) with phenylmethylsulfonyl fluoride (20 μg/ml), leupeptin (0.5 μg/ml), sodium orthovanadate (20 μM), and dithiothreitol (0.1 mM), followed by brain removal. All buffers used for protein extraction contained protease inhibitor cocktail III (#535140, Millipore) and Halt phosphatase inhibitor (#78420, Thermo Fisher Scientific). Hemibrain tissues were weighed and manually homogenized using a dounce homogenizer 1:10 (w/v) in 1XPBS. After homogenization, lysates were centrifuged at 20,800 *g* for 30 minutes at 4 °C. The soluble fraction (supernatant) was removed, and the pellet was resuspended by pipetting in radioimmunoprecipitation assay (RIPA) buffer [50 mM tris, 0.15 M NaCl, 1% octylphenoxypolyethoxyethanol (IGEPAL), 1 mM EDTA, 1 mM EGTA, 0.1% SDS, and 0.5% sodium deoxylate at pH 8] to incubate on ice for 30 minutes. The samples were sonicated (Misonix XL-2000) for 20 seconds on ice, and then centrifuged at 20,800 *g* for 30 minutes at 4 °C. The RIPA-soluble fraction (supernatant) was removed, and the pellet was resuspended in 5 M guanidine (pH 8.0) for analysis of insoluble proteins. Each sample was sonicated for 20 seconds on ice, rotated for 1 hour at room temperature, and then centrifuged at 20,800 *g* for 30 minutes at 4 °C. The bicinchoninic acid (BCA) assay (#23225, Thermo Fisher Scientific) was used to measure the protein concentration of each sample.

For western blots, 20 μg of protein of each sample was loaded onto 4–12% NuPAGE (Invitrogen) gels and separated using MOPS buffer under reduced and denatured conditions. The proteins were transferred onto polyvinylidene difluoride membrane and developed using Pierce ECL (enhanced chemiluminescence; Thermo Fisher Scientific). The developed signals were visualized using ProteinSimple FCR (FluorChem R) and the subsequent chemiluminescent signals were quantified using AlphaView software (ProteinSimple). All blots were analyzed using the Local Background Correction tool except for the HDJ-2 blot, which was analyzed using the Background Link tool in conjunction with Multi-regional Background Correction (AlphaView software).

### Aβ42 ELISA

To measure Aβ42 in hemibrain homogenates from 5XFAD mice, a commercially available Enzyme- Linked-Immunosorbent-Assay (ELISA) (Thermo Fisher Scientific, KHB3441) was used following the manufacturer’s instructions to analyze guanidine and PBS-soluble Aβ42 in hemibrains. To measure Aβ42 in brains of hAPP/PS1 mice, a commercially available ELISA was used (h amyloid β42 ELISA high sensitive, The Genetics Company, Zurich, Switzerland). The ELISA was performed according to the manufacturer’s protocol.

### Mouse brain tissue immunofluorescence

After perfusion, brains were extracted and hemibrains were fixed in 10% formalin followed by cryopreservation in 30% sucrose solution in 1XPBS. A freezing-sliding microtome was used to harvest 30-μm coronal or sagittal sections. Sections were serially placed in a 12-well plate in a cryoprotective solution (1xPBS, 30% sucrose, and 30% ethylene glycol) and stored at − 20 °C until use. Immunofluorescence staining was performed by first washing sections three times in 1XTBS and then incubating sections in 16 mM glycine in 1XTBS for 1 hour at room temperature. After 3 additional washes in 1XTBS, sections were blocked in 5% goat serum in 0.25% Triton X-100 in 1XTBS for 2 hours at room temperature. The sections were then incubated overnight in primary antibodies in a solution of 0.25% Triton X-100, 1% bovine serum albumin and 1XTBS at 4 °C. Secondary immunostaining was then performed with donkey AlexaFluor–labeled secondary antibodies at a concentration of 1:1000 (Thermo Fisher Scientific). ProLong Gold (#P36934, Thermo Fisher Scientific) was used to mount sections before being imaged on a Ti2 wide-field microscope or a Nikon A1 laser scanning confocal microscope for image quantification using Nikon NIS Elements software for acquisition. All acquisition settings remained the same between genotypes and all images were acquired within same imaging period for individual experiments (Northwestern University Center for Advanced Microscopy and Nikon Imaging Centre).

### Image quantification

#### Mouse brains

Immunofluorescence quantification of mouse brain tissue immunostaining was performed on 3–5 sections per animal, from Bregma coordinates of approximately − 0.94 to − 2.55 mm, which were imaged using a 10X objective on a Ti2 wide-field microscope. Quantification analyses, including size and intensity thresholds, were performed using Nikon NIS-Elements Software (Northwestern University Nikon Imaging Center). Depending on the signal, thresholds were set using the General Analysis tool based on object size, shape and contrast, and then binary masks were created for each channel and region of interest. Thresholding was performed separately on each channel to isolate signals of interest by optimizing the signal-to-noise ratio and eliminating non-specific background signal. For the calculation of LAMP1, BACE1, or LysoTracker-Green covered area, as well as LAMP1 and Lysosensor fluorescence intensity measurements in primary neurons, regions of interest were manually traced using NIS-Elements and a binary layer was created for each region of interest. To calculate the ratio of LAMP1 to Aβ42, the area of LAMP1 in dystrophic neurites was normalized to the area of Aβ42 on an individual plaque basis and the average ratios per mouse between treatment groups were quantified. Pearson’s correlation coefficient analysis for BACE1 and LAMP1 was performed using NIS-Elements on a per plaque basis on maximum intensity projections of 30 μm Z-stack images taken using a Nikon A1 laser scanning confocal microscope with a 1 μm step size. To calculate the ratio of LysoTracker-Green to Thiazine red, the area of LysoTracker-Green in dystrophic neurites was normalized to the area of Thiazine Red on an individual plaque basis and the average ratio per mouse was quantified between treatment groups. Four coronal sections from Bregma coordinates of approximately − 1.70 to − 2.55 mm were used to calculate the area or intensity of each signal in each respective brain region for each mouse. Analysis of plaque covered area and plaque size was performed using the average of five sections per mouse from Bregma coordinates of approximately − 0.94 to − 2.55 mm. Section selection, image acquisition, image tracing and quantifications were performed by a person blinded to treatment groups and genotypes.

#### Primary neurons

For the quantifications of LAMP1 immunostaining in fixed mouse primary neurons, somas were manually traced based on morphology using NIS-Elements and a binary mask was created for each region of interest and channel. Thresholding was used to optimize LAMP1 signals and distinguish LAMP1 in neurites, including both dendrites and axons, by isolating pixels of LAMP1 positive for tubulin pixels. The cell soma LAMP1 signals were excluded from total LAMP1 area to analyze LAMP1 vesicles in neurites. For analysis of LysoTracker-Green motility in neurons, images were quantified using NIS-Elements. The speed and distance of total LysoTracker-Green particles in each frame were tracked using the automated motion tracking feature in NIS-Elements. A threshold was set and applied to all movies based on size and fluorescence intensity of LysoTracker-Green particles to eliminate background noise. For kymograph generation, time-lapse imaging of single neurons was performed on a 60X confocal microscope and axons were identified morphologically. The percentage of frequency of particles moving in an anterograde or retrograde direction, or stationary, was quantified by dividing the number of particles for each group by the total number of particles. For kymograph analyses, moving particles were defined as having speed above 0.1 μM/sec.

### Statistical analysis

GraphPad Prism software v8 was used to perform statistical analyses, including analysis of variance (ANOVA) with posthoc tests when comparing more than two samples and unpaired *t* test when only two samples were being compared. Specific test details including number of replicates, type of posthoc tests, and *P* values are stated in figure legends. All quantifications are plotted at the mean ± SEM. Significance was concluded when the *P* value was less than 0.05, indicated by *P < 0.05, **P < 0.01, ***P < 0.001, ****P < 0.0001.

## Results

### Reduced amyloid plaque burden and tau hyperphosphorylation in the brains of LNK-754- chronically treated 5XFAD mice

LNK-754 and lonafarnib are small-molecule FTIs that have been previously tested in humans in cancer clinical trials and are known to cross the blood-brain barrier [8, 9, 41, 42]. α-synuclein [8] and tau inclusions [9] are reduced by LNK-754 and lonafarnib, respectively, but they have not yet been assessed against the amyloid phenotype of AD. In this study, we investigated their effects on amyloid pathology in the 5XFAD mouse model. 5XFAD mice develop Aβ deposits beginning at 2 months of age [36, 37], and dystrophic neurites are observed at the earliest stages of amyloid deposition [39, 43]. Therefore, to coincide with the development of amyloid pathology in 5XFAD mice, we treated mice chronically for 3 months with vehicle, LNK-754, and lonafarnib, beginning at 2 months of age. LNK-754 and lonafarnib were dissolved in a vehicle of 0.5% sodium carboxymethylcellulose and i.p injected daily with a 1 mg/kg dose [8, 44].

**Fig. 1:**
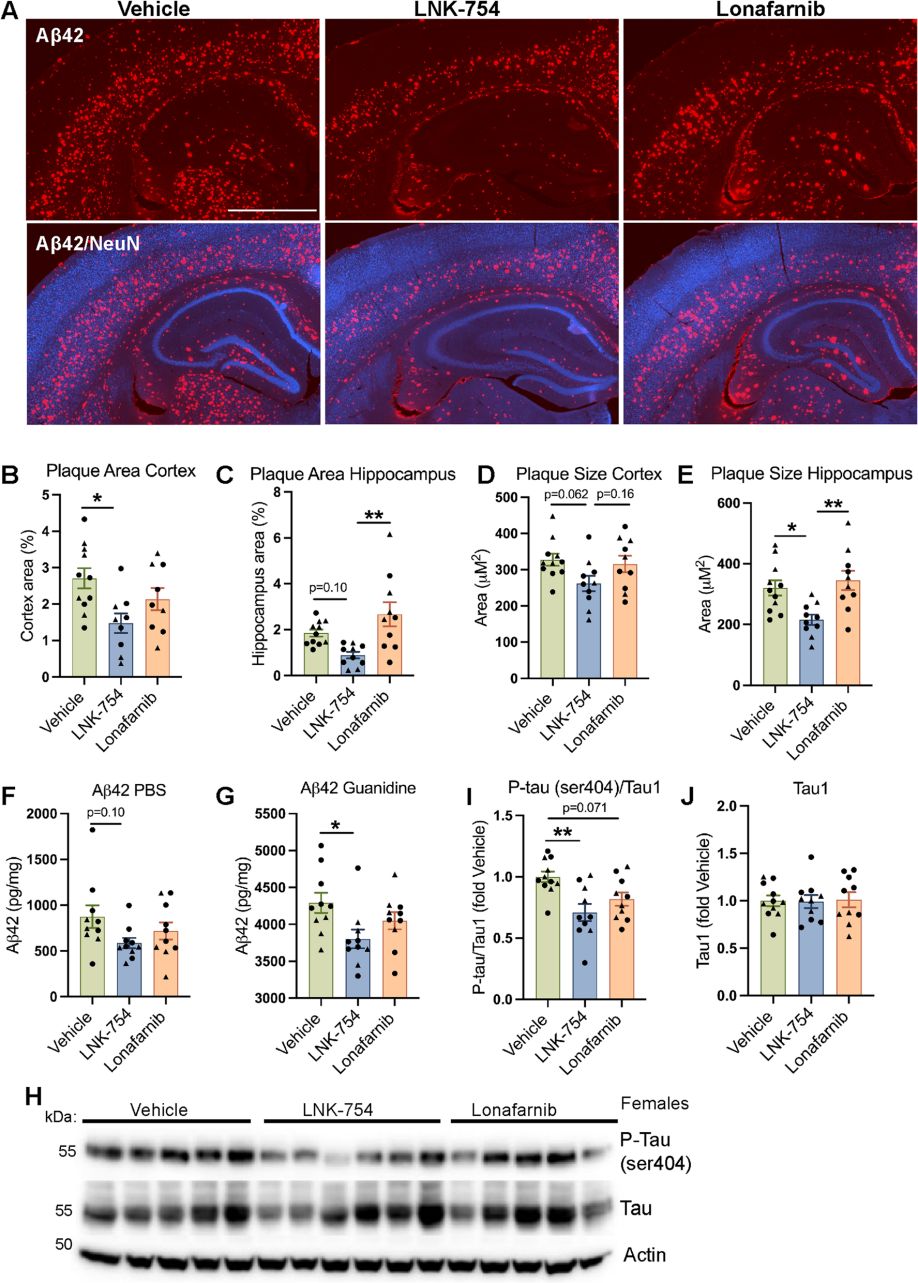
Chronic treatment with FTI LNK-754 reduces amyloid burden and phosphorylated tau in the brains of 5XFAD mice. **A** Confocal immunofluorescence microscopy showing brain sections from 5-month-old 5XFAD mice treated with vehicle, LNK-754 or lonafarnib immunostained for Aβ42 (red) and NeuN (blue). Scale bar, 1000 μm. Quantification of plaque covered area in the cortex (*p = 0.013) **(B)** and the hippocampus (**p = 0.0017) **(C)** in vehicle, LNK-754 or lonafarnib treated 5XFAD mice. Quantification of plaque size in the cortex **(D)** and the hippocampus (*p = 0.017 between vehicle and LNK-754, **p = 0.0036 between LNK-754 and lonafarnib) **(E)** in vehicle, LNK-754 or lonafarnib treated 5XFAD mice. PBS **(F)** and guanidine (*p = 0.028) **(G)**-soluble Aβ42 from hemi-brain homogenates of 5-month-old 5XFAD mice treated with vehicle, LNK-754 or lonafarnib mice was measured by ELISA and expressed as pg/mg protein. **H** Immunoblot of brain homogenates from female vehicle, LNK-754 and lonafarnib treated 5XFAD mice probed for phospho-tau (P-tau ser404) and total tau (Tau1). Quantification of P-tau (ser404) normalized to total tau (**p = 0.0027) **(I)** and total tau normalized to actin **(J)**. Vehicle, *n* = 11 (5 males, 6 females); LNK-754, *n* = 10 (4 males, 6 females); lonafarnib *n* = 10 (4 males, 6 females). Triangles represent males and circles represent females. 1-way ANOVA with Tukey’s post-hoc multiple comparisons test was performed

We first sought to determine the effects of chronic LNK-754 and lonafarnib treatment on Aβ plaque burden in 5XFAD mice by immunofluorescence microscopy for Aβ42 which detects both diffuse and aggregated amyloid, and Thiazine Red (TR), which detects only fibrillar plaque cores (Fig. 1A; Fig. S1). We found a significant decrease in Aβ42 covered area in cortical brain regions of LNK-754 treated mice compared to vehicle-treated controls (Fig. 1B). In the hippocampus, Aβ42 covered area was significantly decreased in LNK-754 treated mice compared to lonafarnib treated mice, and there was a trend toward a decrease in LNK-754 treated mice compared to vehicle-treatment (Fig. 1C). Aβ42 covered area in lonafarnib treated mice was not significantly different from vehicle treated mice in either the cortex or hippocampus (Fig. 1A, B). Plaque size was decreased in cortical and hippocampal brain regions by LNK-754 treatment compared to vehicle treatment; this difference was statistically significant in the hippocampus (Fig. 1D, E; Fig. S2A, B). A significant difference in plaque size between lonafarnib and LNK-754 treatment was also observed in the hippocampus (Fig. 1D, E; Fig. S2A, B). Plaque size was unchanged in lonafarnib treated mice compared to vehicle treated mice in cortical or hippocampal brain regions (Fig. 1D, E; Fig. S2A, B). Aβ plaque seeding was quantified by analyzing plaque number by TR staining. Plaque number significantly decreased in the cortex, but not the hippocampus, of LNK-754 compared to vehicle treated mice (Fig. S2C, D). Reduced plaque burden was confirmed by measuring Aβ42 by ELISA in PBS and guanidine-soluble hemibrain homogenates (Fig. 1F, G). Similar to our immunofluorescence microscopy results, there was a trend towards less Aβ42 in guanidine and PBS fractions in lonafarnib treated mice, whereas a significantly lower level of Aβ42 in guanidine fractions was achieved with LNK-754 treatment (Fig. 1.F, G).

Since sex-based effects in the 5XFAD mouse model have been reported for steady-state APP levels and plaque burden [45, 46], we performed immunoblots of male and female mice treated with vehicle, LNK-754 or lonafarnib separately for transgenic APP expression (Fig. S3A). Transgenic APP levels were measured by immunoblot with the APP 6E10 antibody, which recognizes only human APP, and were unchanged in male or female mice treated with LNK-754, lonafarnib, or vehicle (Fig. S3B-D). A second defining feature of AD is the presence of hyperphosphorylated tau inclusions in the brain and along with plaque deposition, endogenous mouse tau is hyperphosphorylated in 5XFAD mice [47, 48]. To determine if tau hyperphosphorylation was affected by FTI treatment in an amyloid mouse model, immunoblot analysis was performed for phospho-tau at serine 404 (Fig. 1H; Fig. S3E), an epitope known to be hyperphosphorylated in AD brains and in 5XFAD mice [47, 49]. Here, we found reduced phospho-tau ser404 levels in the brains of lonafarnib and LNK-754 treated mice (Fig. 1I), while total tau level remained unchanged (Fig. 1J; Fig. S3F). Altogether our results reveal that amyloid plaque load and pathogenic tau phosphorylation are reduced in the 5XFAD mouse model by pharmacological treatment with FTIs, although LNK-754 treatment was considerably more potent than lonafarnib.

### Chronic treatment with LNK-754 inhibits protein farnesylation in the brains of 5XFAD mice

To confirm that targeting FTase with LNK-754 and lonafarnib by i.p. injection inhibited protein farnesylation in the brains of 5XFAD mice, we evaluated various FTase substrates and downstream markers of FTase inhibition in hemibrain homogenates of 5XFAD mice chronically treated with vehicle, LNK-754 or lonafarnib by immunoblot analysis. We first analyzed prelamin A, which accumulates when FTase is inhibited [50]. An increase in prelamin A was observed in the brains of mice treated with LNK-754 compared with vehicle, while no change occurred in lonafarnib treated mice (Fig. S4A, D). We next looked at HDJ-2, a chaperone protein that undergoes an upward mobility shift following FTI treatment and is widely used as a marker of FTase inhibition [50]. Consistent with prelamin A, a slower migrating form of HDJ-2 was present in LNK-754 treated mice but not in lonafarnib treated mice (Fig. S4B, E). A significant increase in the slower migrating species of HDJ-2 was found in LNK-754 mice compared to vehicle or lonafarnib treated mice (Fig. S4E). In addition, farnesylation activates Ras, so we also measured the phosphorylation of its downstream effector molecule ERK1 (P44/P42) (Fig. S4C). Whereas HDJ-2 and prelamin A were unchanged by lonafarnib treatment, a trend towards reduced pERK1 was observed (Fig. S4F). pERK1 was also decreased in mice treated with LNK-754 (Fig. S4F). Our results suggest that 1 mg/kg LNK-754 inhibited FTase in the brain, however lonafarnib was less effective. This could be due to the lower potency and IC50 of lonafarnib as shown previously [41, 51] or possibly reduced brain exposure.

### Decreased LAMP1 accumulation in dystrophic neurites in LNK-754 chronically treated 5XFAD mice

In healthy neurons, lysosomal components are delivered to endosomes and autophagic organelles in distal neurites, which become acidified and mature during retrograde trafficking toward the neuronal soma [52, 53]. Amyloid plaques disrupt this process, resulting in dystrophic neurites that entrap dysfunctional autophagic and endolysosomal organelles. Dystrophic neurites block the axonal transport of BACE1, leading to Aβ generation within dystrophic neurites and extracellular plaque deposition [16–18]. FTIs are known to enhance lysosomal function and microtubule stability, so to further investigate how FTIs reduce plaque burden, we focused specifically on dystrophic neurites in 5XFAD mice.

**Fig. 2:**
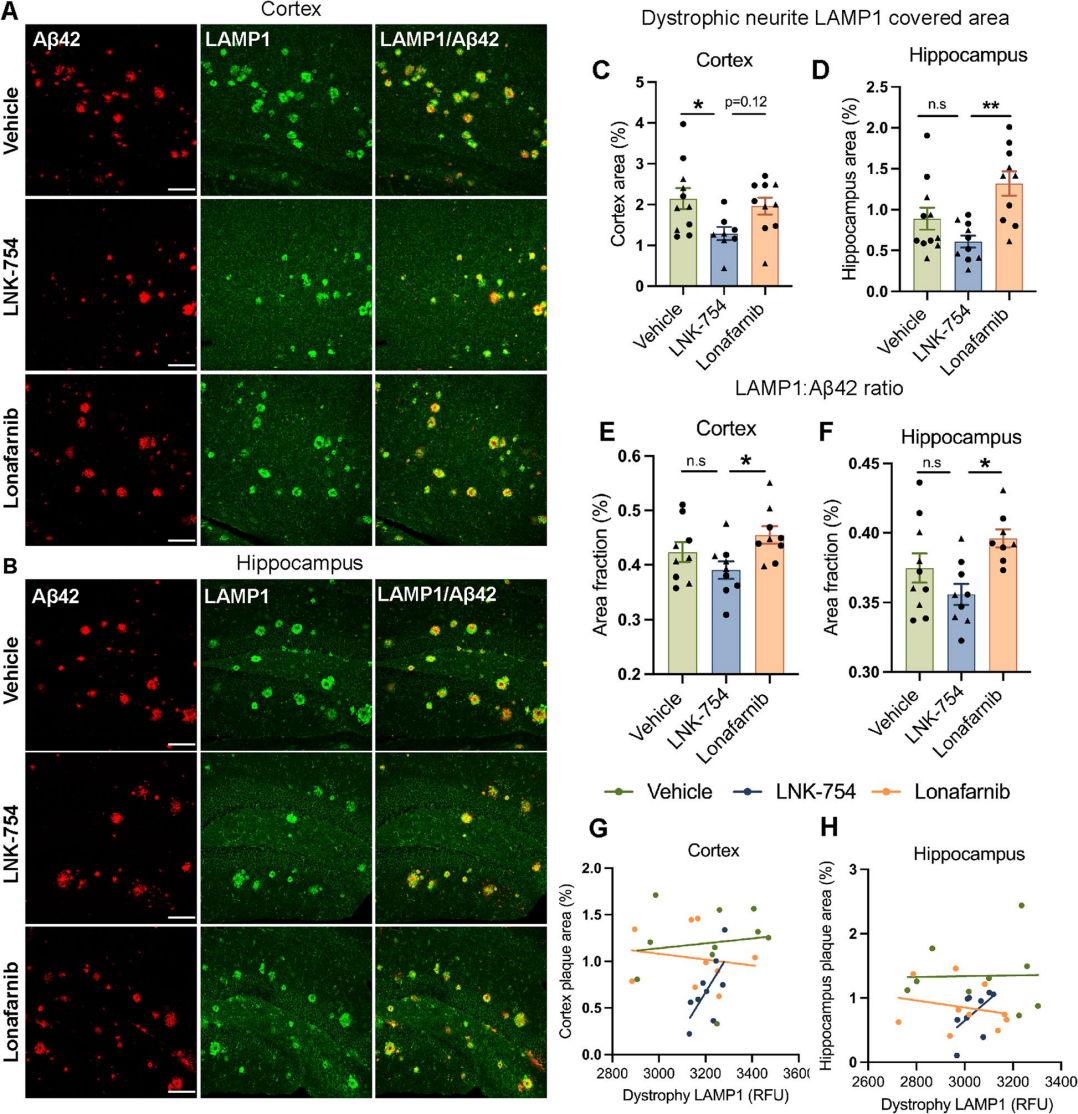
Decreased levels of LAMP1 in dystrophic neurites is associated with reduced plaque burden in LNK-754-chronically treated 5XFAD mice. Confocal immunofluorescence microscopy showing cortical **(A)** and hippocampal regions **(B)** from 5-month-old 5XFAD mice treated with vehicle, LNK-754, and lonafarnib immunostained for Aβ42 (red) and LAMP1 (green). Scale bar, 100 μm. Quantification of LAMP1 covered area in dystrophic neurites the cortex (*p = 0.037) **(C)** and hippocampus (**p = 0.0012) **(D)** in vehicle, LNK-754 and lonafarnib treated 5XFAD mice. Quantification of the ratio of dystrophic neurite LAMP1 covered area normalized to plaque area in cortical (*p = 0.030) **(E)** and hippocampal (*p = 0.011) **(F)** brain areas. Correlations of plaque area (Fig. 1) in the cortex **(G)** and hippocampus **(H)** plotted against LAMP1 fluorescence intensity in dystrophic neurites (Fig. S5E, F). Vehicle, *n* = 11 (5 males, 6 females); LNK-754, *n* = 10 (4 males, 6 females); lonafarnib *n* = 10 (4 males, 6 females). Triangles represent males and circles represent females. 1-way ANOVA with Tukey’s post-hoc multiple comparisons test was performed in C-F and linear regression was performed in G and H. Vehicle; Rsq = 0.0170, *p* = 0.719, LNK-754; Rsq = 0.699, *p* = 0.0097, Lonafarnib; Rsq = 0.0521, *p* = 0.526 in **(G)**. Vehicle; Rsq = 0.0527, *p* = 0.524, LNK-754; Rsq = 0.289, *p* = 0.141, Lonafarnib; Rsq = 0.0006, *p* = 0.9495 in **(H)**

To quantify the accumulation of dystrophic neurites around plaques, we evaluated lysosomal-associated membrane protein 1 (LAMP1), a marker of heterogenous endolysosomal and autophagic organelles. Hippocampal and cortical brain tissues from 5XFAD mice treated with vehicle, LNK-754 and lonafarnib were co-stained for LAMP1 and Aβ42 and confocal immunofluorescence microscopy was performed (Fig. 2A, B). A threshold was set to eliminate LAMP1 fluorescence signals from vesicles in healthy neurites and somas to specifically analyze dystrophic neurites. LAMP1 covered area was significantly decreased in cortical and hippocampal brain regions of LNK-754 treated mice (Fig. 2C, D), whereas no difference was observed in lonafarnib treated mice compared to vehicle treatment (Fig. 2C, D). The deposition of amyloid plaques initiates the development of dystrophies in nearby neurites, mainly axons and presynaptic terminals. Correspondingly, the effects of LNK-754 and lonafarnib on dystrophic neurites are consistent with their effects on plaque burden (Fig. 1). Additional analysis of the effect of FTI treatment on dystrophic neurites was performed by quantifying the ratio of LAMP1 covered area to Aβ42 area to determine whether FTIs constrain dystrophy size around plaques. A decrease in the ratio of LAMP1: Aβ42 was observed for LNK-754 treated mice compared to vehicle mice, and LNK-754 and lonafarnib treatments were significantly different (Fig. 2E, F), indicating that LNK-754 mice associated with fewer dystrophic neurites.

Elevated levels of LAMP1 were found in hemibrain homogenates of mice treated with either LNK-754 or lonafarnib (Fig. S5A, B), consistent with previous studies showing that lonafarnib and LNK-754 increase the number of autolysosomes and levels of lysosomal membrane proteins and hydrolases [8, 9]. In agreement with immunoblot analysis results for LAMP1 (Fig. S5A, B), the mean fluorescence intensity of total LAMP1 immunostaining was significantly increased in the brains of LNK-754 and lonafarnib treated mice compared with vehicle treated mice (Fig. S5C, D). In contrast to the total LAMP1 fluorescence level, the mean LAMP1 fluorescence intensity in dystrophic neurites was unchanged between vehicle, LNK-754 and lonafarnib treated mice (Fig. S5E, F). In the cortex of LNK-754 treated mice, LAMP1 fluorescence intensity in dystrophies and plaque burden were highly correlated, while no correlation between LAMP1 intensity in dystrophies and plaque burden was found in lonafarnib or vehicle treated mice (Fig. 2G, H), revealing a connection between dystrophic neurite LAMP1 accumulation and plaque burden in LNK-754 mice. A trend towards a correlation between LAMP1 intensity in dystrophies and plaque burden was observed in the hippocampus. Together, our results demonstrate that LNK-754 treatment increases the total level of LAMP1 yet attenuates the pathological accumulation of LAMP1 in dystrophic neurites that is usually present in 5XFAD mice.

### Decreased BACE1 accumulation in dystrophic neurites in LNK-754 chronically treated 5XFAD mice

**Fig. 3:**
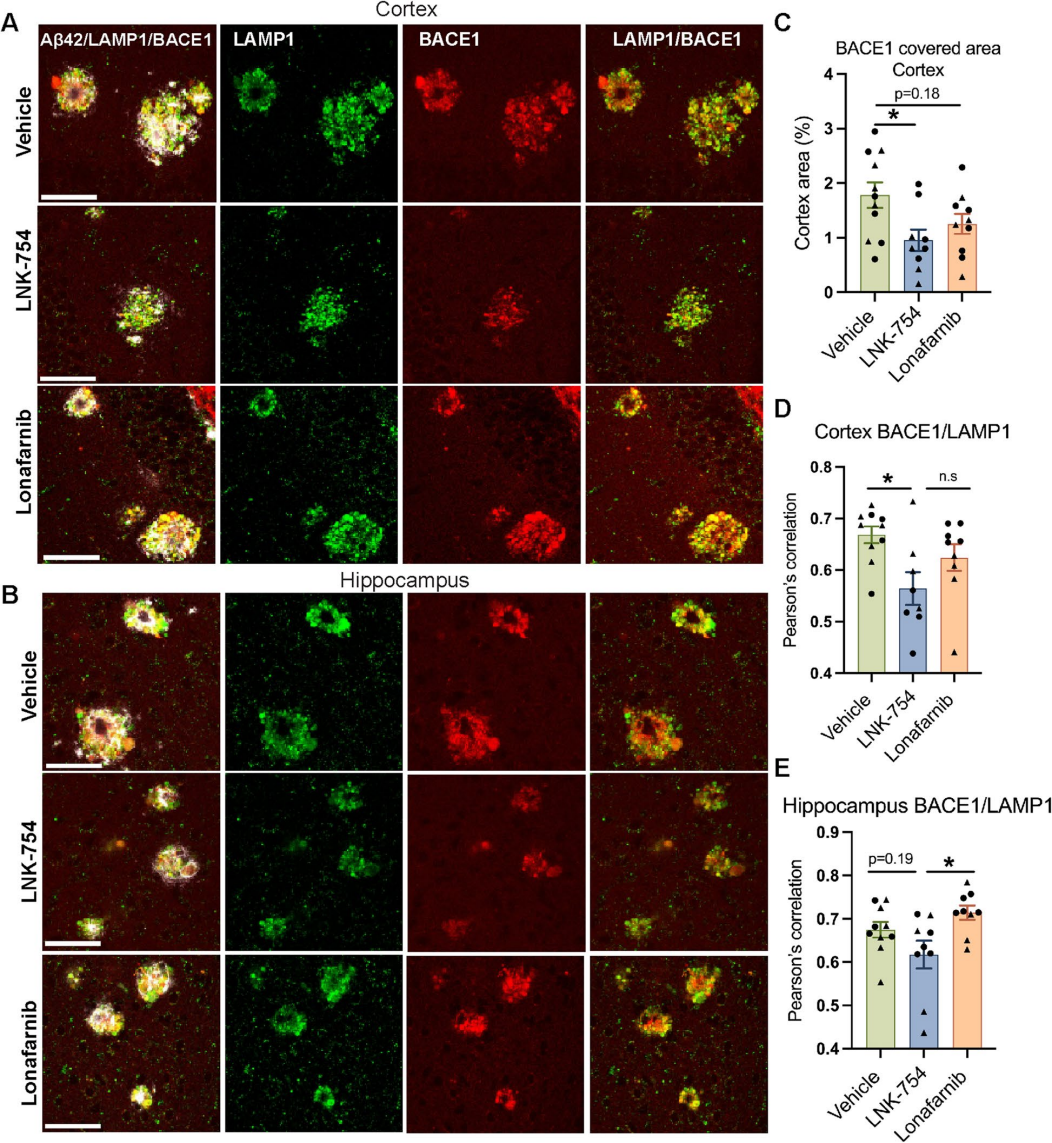
Chronic treatment with LNK-754 decreases BACE1 in dystrophic neurites in 5XFAD mice. Confocal immunofluorescence microscopy showing cortical **(A)** and hippocampal **(B)** regions from 5-month-old 5XFAD mice treated with vehicle, LNK-754, and lonafarnib immunostained for Aβ42 (white), LAMP1 (green) and BACE1 (red). Scale bar, 50 μm. Quantification of BACE1 covered area in the cortex (*p = 0.026) **(C)**. Quantification of BACE1 and LAMP1 co-localization in the cortex (*p = 0.017) **(D) (**Vehicle, 188; LNK-754, 177; lonafarnib,168 plaques analyzed) and hippocampus (*p = 0.018) **(E) (**Vehicle, 87; LNK-754, 68; lonafarnib, 108 plaques analyzed). Vehicle, *n* = 11 (5 males, 6 females); LNK-754, *n* = 10 (4 males, 6 females); lonafarnib *n* = 10 (4 males, 6 females). Triangles represent males and circles represent females. 1-way ANOVA with Tukey’s post-hoc multiple comparisons test was performed

LAMP1 is found on membranes of endolysosomal organelles that target axonal BACE1 to mature lysosomes for degradation. Therefore, FTIs could inhibit Aβ generation and extracellular plaque deposition by reducing axonal dystrophy formation and the accumulation of BACE1 around plaques. In support of this hypothesis, dystrophic neurite LAMP1 level and plaque burden were highly correlated in cortical brain regions in LNK-754 mice. Our analysis of LAMP1 immunostaining revealed that, compared to vehicle or lonafarnib treatment, plaques in LNK-754 treated 5XFAD mice were associated with fewer dystrophic neurites (Fig. 2). To determine whether the level of BACE1 in dystrophic neurites was similarly decreased per plaque, we performed confocal immunofluorescence microscopy of cortical and hippocampal brain tissues of vehicle, LNK-754 and lonafarnib treated mice for BACE1, in addition to LAMP1 and Aβ42 (Fig. 3A, B). Here, BACE1 covered area was significantly decreased in cortical brain regions of LNK-754 treated mice, and a trend was observed in lonafarnib treated mice (Fig. 3C). The analysis of BACE1 covered area in dystrophic neurites in the hippocampus was impeded by the high expression level of BACE1 in presynaptic terminals of the CA3 hippocampal mossy fiber pathway [17]. We additionally analyzed whether less BACE1 localized to endolysosomes in dystrophic neurites by measuring Pearson’s correlation coefficient for BACE1 and LAMP1 in dystrophies. A significant decrease in BACE1 and LAMP1 co-localization per plaque was found in cortical brain regions in LNK-754 treated mice compared to vehicle treatment (Fig. 3D). In the hippocampus, a significant decrease in BACE1 and LAMP1 co-localization per plaque was found for LNK-754 compared to lonafarnib, and a trend towards a decrease was observed for LNK-754 treatment compared to vehicle (Fig. 3E). These results are consistent with our findings for plaque burden and LAMP1 covered area, and further support the idea that LNK-754 treatment attenuated the formation of dystrophic neurites, reducing the buildup of impaired endolysosomal organelles that target BACE1 for degradation.

### Acute treatment with LNK-754 reduces late endosome and lysosome accumulation in dystrophic neurites in 5XFAD mice live brain slices

**Fig. 4:**
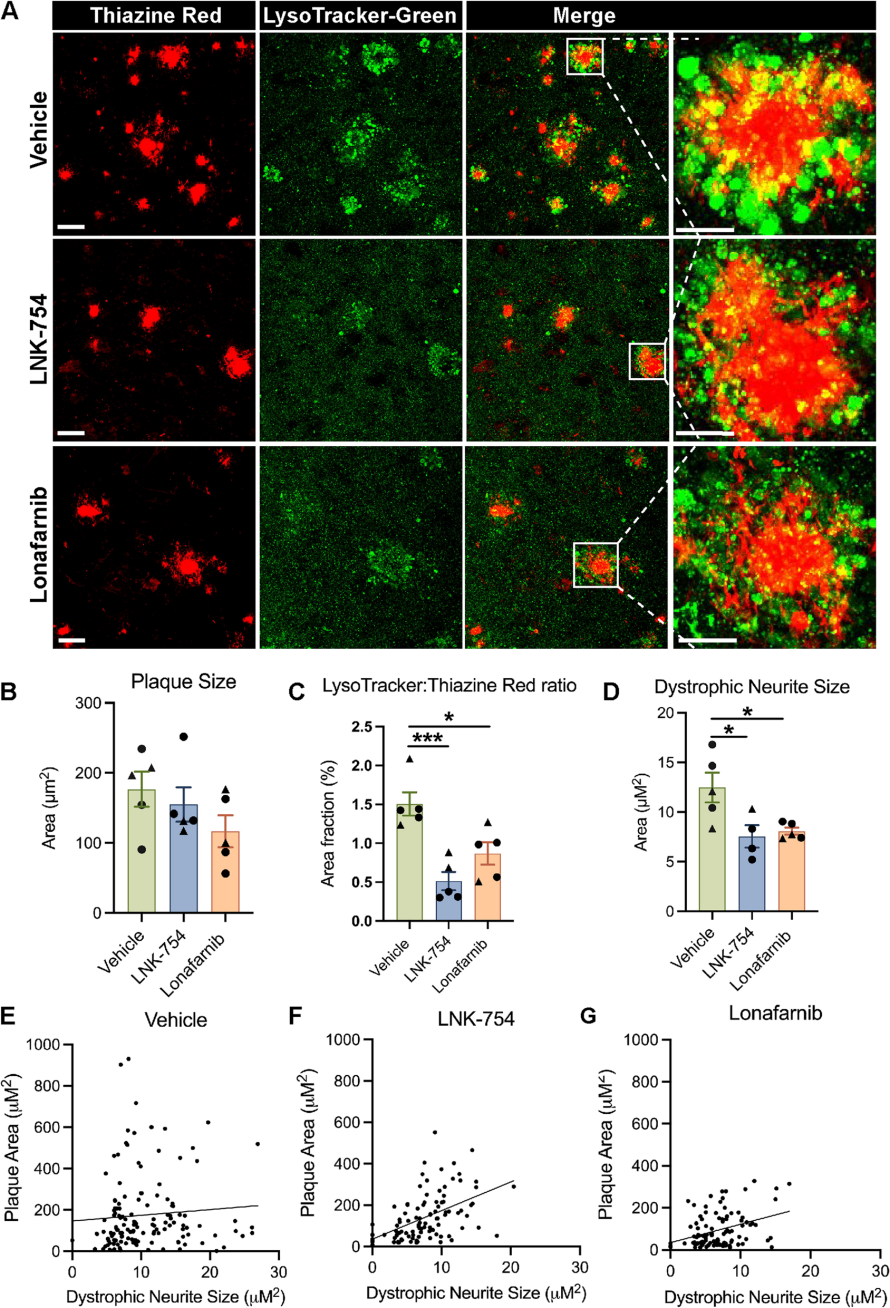
Acute FTI treatment reduces LysoTracker-Green accumulation in dystrophic neurites surrounding plaques in 5XFAD mice. **A** Confocal immunofluorescence microscopy showing plaques in cortical brain regions from 5-month-old 5XFAD mice treated for 3 weeks with vehicle, LNK-754, or lonafarnib labeled with thiazine red (red) and LysoTracker-green (green). Scale bar, 25 μm; high magnification images, 10 μm. Maximum intensity projections of 30 μm Z-stack images are shown. **B** Quantification of thiazine red covered area for individual plaques in vehicle, LNK-754 or lonafarnib treated 5XFAD mice. **C** Quantification of LysoTracker-green covered area in dystrophic neurites normalized to thiazine red area per plaque in vehicle, LNK-754 and lonafarnib treated 5XFAD mice (***p = 0.0007 between vehicle and LNK-754, *p = 0.017 between vehicle and lonafarnib). **D** Quantification of the average size of dystrophic neurites, measured by quantifying average object area of LysoTracker-Green in dystrophic neurites per plaque (*p = 0.026 between vehicle and LNK-754, *p = 0.035 between vehicle and lonafarnib). Correlations of plaque area plotted against LysoTracker-Green object area in vehicle (**E**) LNK-754 (**F**) and lonafarnib (**G**) mice. Vehicle, Rsq = 0.0062, *p* = 0.3482; LNK-754, Rsq = 0.2664, *p* < 0.0001; Lonafarnib, Rsq = 0.1322, *p* = 0.0002. Vehicle, *n* = 5 (2 males, 3 females); LNK-754, *n* = 5 (2 males, 3 females); lonafarnib *n* = 5 (2 males, 3 females). Triangles represent males and circles represent females. 1-way ANOVA with Tukey’s post-hoc multiple comparisons test was performed in B-D and linear regression was performed in E-G

We next tested the acute effects of LNK-754 and lonafarnib on dystrophic neurites by performing confocal imaging of live brain tissues. In this experiment, 5-month-old 5XFAD mice were injected daily with vehicle, LNK-754 or lonafarnib for 3 weeks. We stained tissues with LysoTracker-Green (LT), a probe commonly used for tracking and selectively identifying acidified late endosomes and lysosomes in living cells [53]. The pattern of LT and thiazine red staining was consistent with our immunostaining results for dystrophic neurite markers in 5XFAD mice (Fig. 2A, B; Fig. 3A, B); LT-positive late endosomes and lysosomes were highly concentrated in dystrophic neurites closely associated with amyloid plaques in living brain tissues (Fig. 4A). An acute 3-week treatment of 5-month-old 5XFAD mice with LNK-754 or lonafarnib was insufficient to reduce plaque size (Fig. 4B), although LNK-754 treatment caused a significant decrease in the ratio of LT: TR per plaque and dystrophic neurite size (Fig. 4C, D). Interestingly, in contrast to our findings for LAMP1 or BACE1, acute lonafarnib treatment also decreased dystrophy size and the ratio of LT: TR per plaque (Fig. 4C, D). Dystrophic neurite size and plaque size highly correlated in LNK-754 treated 5XFAD mice (Fig. 4F), and a significant correlation was also observed in lonafarnib treated mice (Fig. 4G). No correlation existed in vehicle treated mice (Fig. 4E). Our results confirm that LNK-754 decreased axonal dystrophy and suggest that FTIs can attenuate late endosome and lysosome accumulation around plaques, even after extensive plaque deposition and axon degeneration.

### Improvement of learning and memory deficits in hAPP/PS1 mice after short-term treatment with LNK-754

**Fig. 5:**
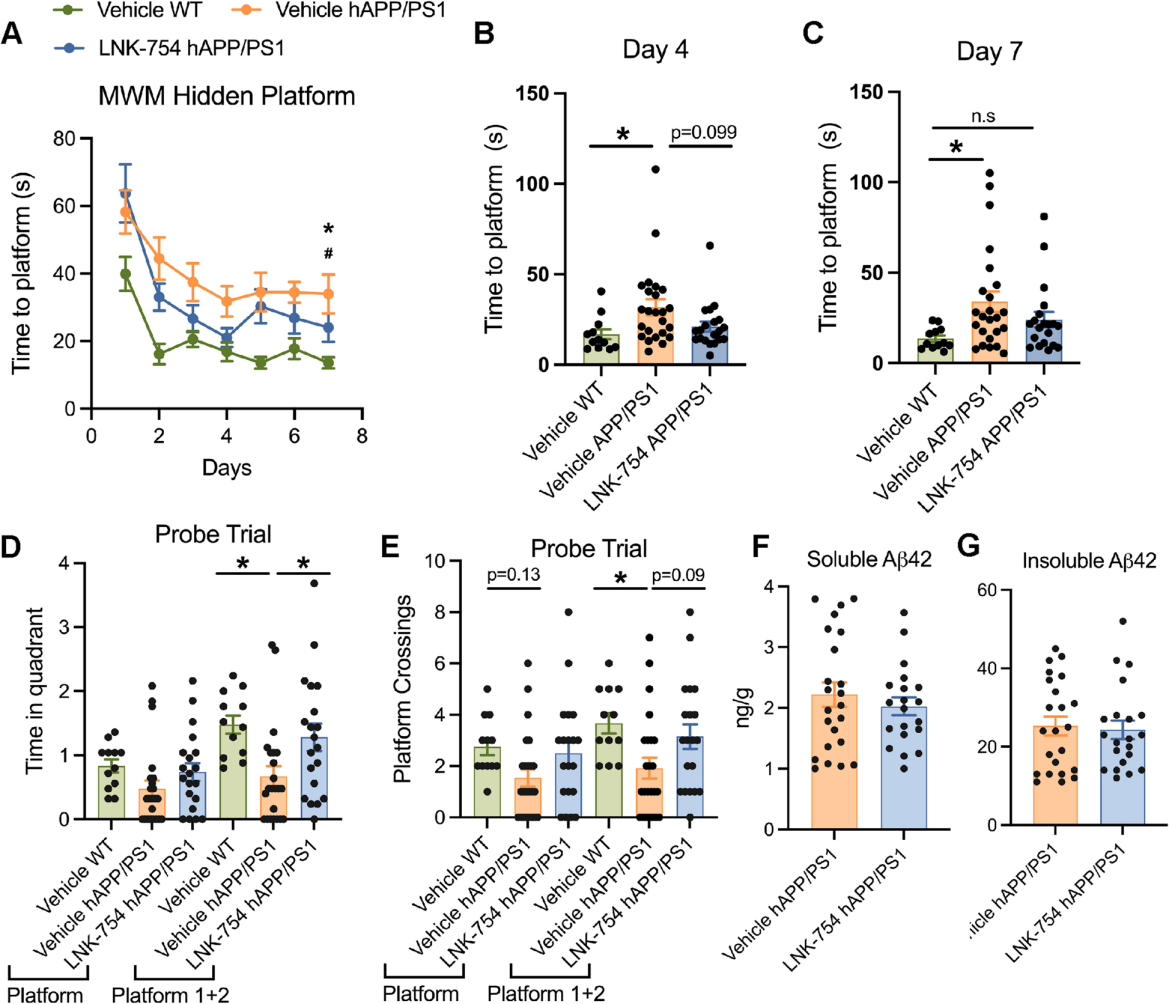
LNK-754 improves learning and memory deficits in hAPP/PS1 mice. Female 6-month-old vehicle-treated wild-type mice, vehicle-treated hAPP/PS1 mice and LNK-754-treated hAPP/PS1 mice were trained in the Morris Water Maze (MWM) using a hidden platform and analyzed for the time to reach the platform in seven consecutive trials **(A)**. # *p* < 0.01, LNK-754 treated hAPP/PS1 mice versus vehicle-treated hAPP/PS1 mice in the learning curve of the MWM, * *p* < 0.05 vehicle treated hAPP/PS1 mice versus wild-type controls in the learning curve of the MWM. Individual level data is shown for days 4 (*p = 0.040) **(B)** and 7 (*p = 0.030) **(C)**. 24 hours after the last training day, each animal had a probe trial with the platform removed assessing the time spent in the hidden platform quadrant (*p = 0.016 between vehicle-treated wild-type and vehicle-treated hAPP/PS1, *p = 0.038 between vehicle-treated hAPP/PS1 and LNK-754 treated hAPP/PS1) **(D)** and the number of platform crossings (*p = 0.036 between vehicle-treated wild-type and vehicle-treated hAPP/PS1) **(E)**. Platform 1 refers to the location of the original platform, platform 1 and 2 refers to the location of the original platform and a one-inch perimeter. Soluble **(F)** and insoluble **(G)** Aβ42 from brain homogenates of 6-month-old vehicle-treated wild-type mice, vehicle-treated hAPP/PS1 mice and LNK-754-treated hAPP/PS1 mice (vehicle-treated wild-type *n* = 12, vehicle-treated hAPP/PS1 *n* = 24, LNK-754-treated hAPP/PS1 mice *n* = 20). Dunnett’s multiple comparison test, unpaired t-test and 1-way ANOVA with Tukey’s post-hoc multiple comparisons test were performed

LNK-754 prevented the accumulation of LAMP1, BACE1 and LT in dystrophic neurites in 5XFAD mice, suggesting that LNK-754 treatment protects against Aβ-associated synaptic dysfunction. To evaluate whether LNK-754 treatment had a beneficial effect on cognitive function, we assessed the short-term efficacy of oral LNK-754 treatment on spatial learning and memory in the hAPP/PS1 AD mouse model [40]. Spatial memory of hAPP/PS1 mice was improved after 12 days of oral treatment with LNK-754, as assessed by the Morris Water Maze (MWM). During the hidden platform training sessions, LNK-754 treated hAPP/PS1 mice learned to locate the platform faster than vehicle treated hAPP/PS1 mice, which resulted in a learning curve that is significantly different from that of vehicle-treated hAPP/PS1 control mice (Fig. 5A). Vehicle-treated hAPP/PS1 mice took significantly more time to locate the platform compared to vehicle-treated wild-type controls on days 4 and 7 of the training trials, whereas no significant differences were found between LNK-754 treated hAPP/PS1 mice and vehicle-treated wild-type mice (Fig. 5A-C). During the probe test, LNK-754 treated hAPP/PS1 animals showed increased time spent in the target quadrant (Fig. 5D) and increased frequency of platform crossings (Fig. 5E), comparable to vehicle-treated wild-type animals. Aβ42 levels were measured by ELISA and were unchanged in soluble and insoluble brain homogenates from vehicle treated or LNK-754 treated hAPP/PS1 mice (Fig. 5F, G), suggesting that improved memory and spatial learning were not a result of lowered amyloid burden in LNK-754 treated hAPP/PS1 mice.

### LNK-754 and lonafarnib enhance axonal endolysosomal trafficking in cultured neurons

**Fig. 6:**
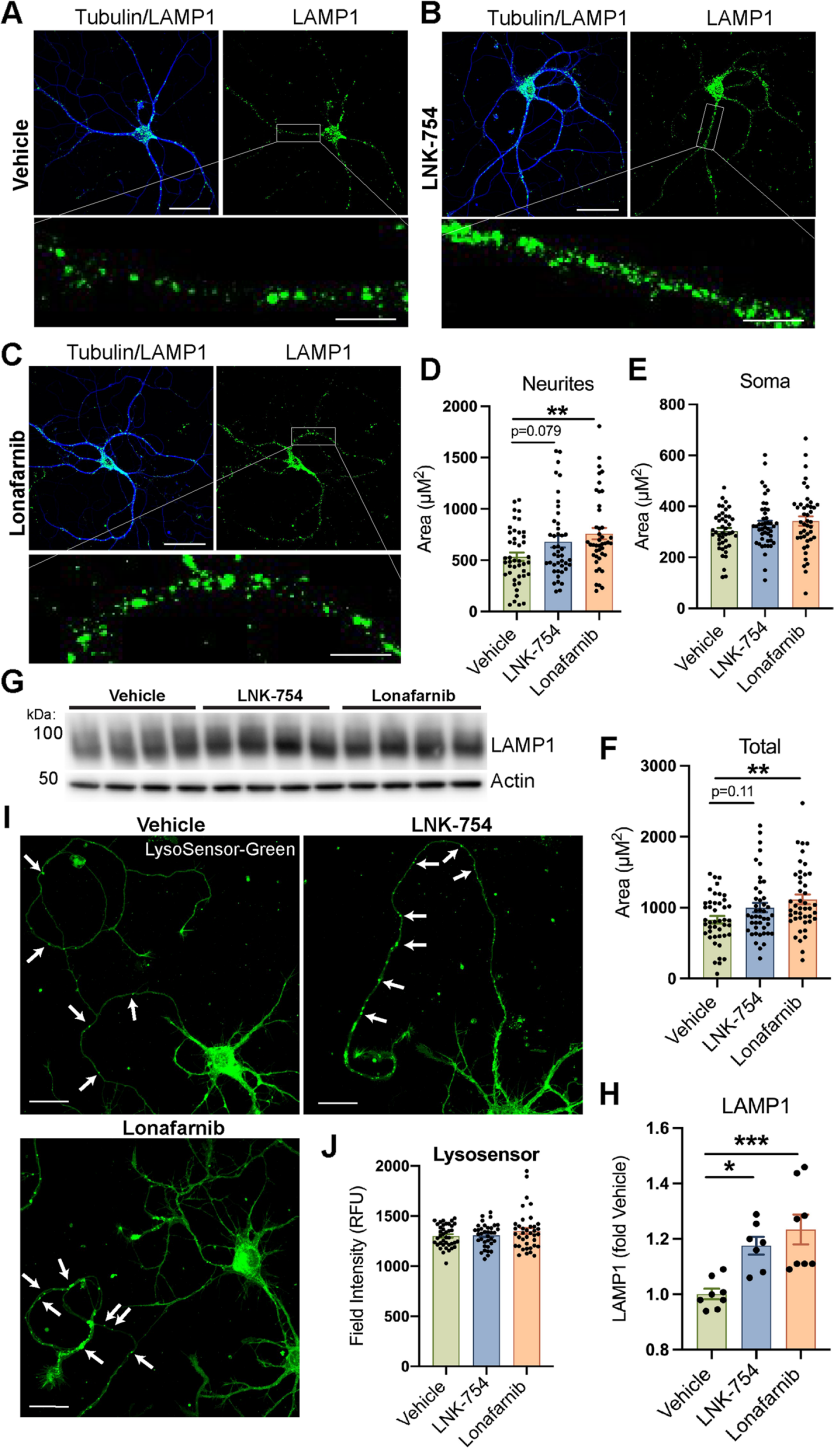
FTI treatment increases LAMP1 in proximal and distal neurites in mouse primary neurons. Wild-type primary forebrain neurons were grown for seven days in culture and treated for 48 hours with vehicle (DMSO), 10 nM LNK-754 or 10 nM lonafarnib. **A-C** Confocal immunofluorescence images showing cells immunostained for LAMP1 (green) and β3-tubulin (blue). Magnified images show LAMP1 in neurites. Scale bars, 50 μm, 10 μm (magnified images). Quantification of LAMP1-covered area in cell neurites (**p = 0.0061) (**D)** soma **(E)** and total area (**p = 0.0045) **(F)**. *N* = 3 independent experiments, values from individual cells are plotted. 10–16 cells were analyzed from 2 to 3 coverglasses per treatment group in each experiment. Total number of cells analyzed: vehicle, 44; LNK-754, 43; lonafarnib, 43. **G** Immunoblot of cell lysates from vehicle, LNK-754 and lonafarnib treated wild-type primary neurons probed for LAMP1 and actin. **H** Quantification LAMP1 immunoblot in (**G)**. Signals were normalized to actin and expressed as fold of vehicle (*p = 0.013 between vehicle and LNK-754, ***p = 0.0008 between vehicle and lonafarnib). **I** Confocal immunofluorescence images showing cells stained with LysoSensor-Green. Scale bars, 25 μm. Arrows denote LysoSensor vesicles in distal neurites. **J** Quantification of LysoSensor-Green fluorescence intensity. *N* = 3 independent experiments, values from individual cells are plotted. 10–16 cells were analyzed from 2 to 3 coverglasses per treatment group in each experiment. Total number of cells analyzed: vehicle, 39; LNK-754, 36; lonafarnib, 38. 1-way ANOVA with Tukey’s post-hoc multiple comparisons test was performed

To investigate the mechanism of LNK-754 to decrease the accumulation of LAMP1, BACE1 and LT in dystrophic neurites in 5XFAD mice, we performed in vitro studies using wild-type mouse primary cultured neurons. We first evaluated the distribution pattern of endolysosomal organelles in neurons treated with vehicle, LNK-754 or lonafarnib by confocal immunofluorescence microscopy. In vehicle-treated neurons, LAMP1 fluorescent vesicles were concentrated mostly in cell bodies and proximal neurites, whereas in LNK-754 and lonafarnib treated neurons, LAMP1 vesicles were increased in proximal and distal neurite areas compared to vehicle treated neurons (Fig. 6A-F). Similar to our in vivo results and in agreement with previous reports [9], increased total LAMP1 was also present in LNK-754 and lonafarnib treated cultures (Fig. 6F-H). Lonafarnib treatment significantly increased total LAMP1 and LAMP1 localizing to neurites, whereas a trend was observed for LNK-754 treatment (Fig.6D, F). LAMP1 non-specifically marks various endosomal and autophagic vesicles, so we next analyzed the localization and acidification of late endosomes and lysosomes in live neuron cultures treated with LysoSensor Green, a fluorescent dye that exhibits a pH-dependent increase in fluorescence intensity in acidified compartments. Total LysoSensor Green fluorescence intensity was unchanged in neurons treated with vehicle, LNK-754 or lonafarnib (Fig. 6I, J). Consistent with our findings for LAMP1, the localization of LysoSensor positive-vesicles increased in distal neurites of LNK-754 or lonafarnib treated cultures (Fig. 6I, arrows).

**Fig. 7:**
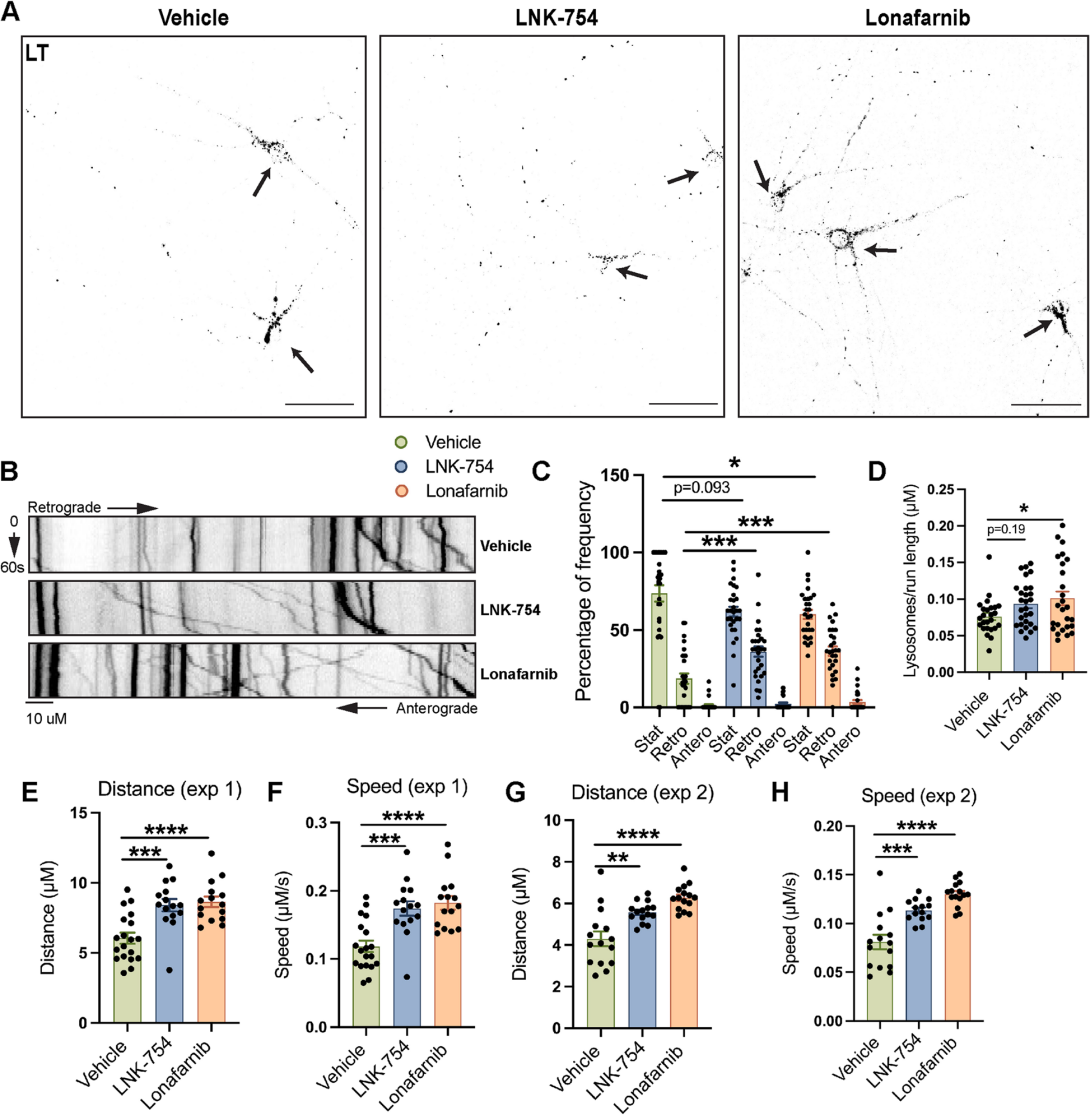
FTI treatment enhances retrograde lysosomal trafficking in mouse primary forebrain neurons. Wild-type primary forebrain neurons were grown for seven days in culture and treated for 48 hours with vehicle (DMSO), 10 nM LNK-754 or 10 nM lonafarnib. **A** Representative images showing Lysotracker Green fluorescence (black puncta) in wild-type primary forebrain neurons. Arrows denote cell bodies. Scale bar, 50 μm. **B** Kymographs of vehicle (*n* = 25), LNK-754 (*n* = 28) and lonafarnib (*n* = 26)-treated wild-type primary forebrain neurons. Quantifications of the frequency distribution of LT vesicle movement (for stationary particles, *p = 0.046 between vehicle and lonafarnib, for retrograde particles, ***p = 0.0007 between vehicle and LNK-754, and ***p = 0.0005 between vehicle and lonafarnib) **(C)** and number of LT vesicles per axon length (*p = 0.040 between vehicle and lonafarnib) **(D)**. Quantifications of LT vesicle distance (in exp. 1, ***p = 0.004 between vehicle and LNK-754, ****p < 0.001 between vehicle and lonafarnib, in exp. 2, **p = 0.0012 between vehicle and LNK-754, ****p < 0.0001 between vehicle and lonafarnib) **(E, G)** and speed (in exp. 1, ***p = 0.0004 between vehicle and LNK-754, ****p < 0.0001 between vehicle and lonafarnib, in exp. 2, ***p = 0.0001 between vehicle and LNK-754, ****p < 0.0001 between vehicle and lonafarnib) **(F, H)** traveled (*n* = 2 experiments, 15–20 replicates analyzed per group). Each data point corresponds to the mean speed or distance of LT particle movement in neurons treated with vehicle, LNK-754 or lonafarnib per replicate in each experiment. 1-way ANOVA with Tukey’s post-hoc multiple comparisons test was performed

A considerable percentage of LAMP1-positive vesicles were localized to proximal and distal neurites in FTI-treated neurons, suggesting that FTase inhibition affects the motility of endolysosomal organelles. We assessed the effects of FTI treatment on the dynamics of late endosomes and lysosomes by time-lapse imaging of live wild-type primary neuronal cultures stained with LT (Fig. 7A). Kymograph analysis of axonal LT vesicles revealed that, under all treatment conditions, most axonal LT vesicles were stationary, or traveled in a low velocity, retrograde direction (Fig. 7B). However, there was a strong shift in the percentage of stationary, retrograde, and anterograde axonal LT vesicles in LNK-754 and lonafarnib treated neurons. The percentage of retrograde LT vesicles in LNK-754 (36%) or lonafarnib (36%) treated neurons was significantly higher than in vehicle treated neurons (19%) while the percentage of stationary LT vesicles in LNK-754 (62%) or lonafarnib (60%) treated neurons was lower compared to vehicle treated neurons (74%) (Fig. 7C). A significant increase in the number of LT vesicles per axon length was observed in lonafarnib treated neurons and a trend was observed for LNK-754 treatment (Fig. 7D), as in the distribution of LAMP1 (Fig. 6A-D) and LysoSensor (Fig. 6I, J). The distance traveled by LT vesicles was significantly increased in LNK-754 and lonafarnib treated neurons (Fig. 7E, G) and the average velocity of LT vesicles was significantly faster in LNK-754 (0.17 μm/s) and lonafarnib (0.18 μm/s) treated neurons than in vehicle (0.11 μm/s) treated neurons (Fig. 7F, H; movie S1, S2, S3). Together with our results from FTI-treated 5XFAD mice, these data support a role of FTase in regulating both the generation and axonal motility of late endosomes and lysosomes.

## Discussion

Many neurodegenerative diseases are characterized by impaired lysosomal systems and the buildup of disease-specific protein aggregates. AD brains in particular show extensive lysosome dysfunction and can be distinguished from other neurodegenerative diseases by the accumulation of autophagic and endolysosomal organelles in enlarged dystrophic neurites surrounding amyloid plaques [54]. FTIs are a promising therapeutic candidate for repurposing towards treating neurodegenerative diseases that share the pathologies of lysosome dysfunction and protein accumulation. By increasing lysosomal function, FTIs could have significant therapeutic effects on dystrophic neurites in the AD brain. However, until now, the impact of FTIs on Aβ pathology and axonal dystrophy had yet to be tested in vivo.

We determined the effect of brain penetrant FTIs LNK-754 and lonafarnib on AD-relevant pathology in the 5XFAD mouse model and present new data showing that chronic treatment of 5XFAD mice with LNK-754 reduced amyloid plaque burden and attenuated dystrophic neurite formation. While not a direct focus of this study, LNK-754 and lonafarnib treatment also decreased tau hyperphosphorylation in the brains of 5XFAD mice. In contrast to our findings for LNK-754, lonafarnib treated 5XFAD mice showed only slight improvements in AD-relevant pathology and consistently, markers of FTase inhibition were mostly unchanged in the brains of lonafarnib mice. LNK-754 and lonafarnib were both effective in vitro; the number and velocity of axonal endolysosomal organelles trafficking in a retrograde direction were dramatically increased in live cultured primary neurons. LNK-754 and lonafarnib also increased levels of endolysosomal marker LAMP1 in primary neuron cultures and in the brains of 5XFAD mice. Thus, FTI treatment stimulated endolysosomal biogenesis and retrograde axonal trafficking of late endosomes and lysosomes in vitro, which may have slowed axon degeneration and inhibited dystrophic neurite formation and plaque deposition in 5XFAD mice. Notably, LNK-754 reduced BACE1 accumulation in dystrophic neurites, and blocked the feed-forward pathway of axonal dystrophy, Aβ42 generation and plaque deposition. Our results agree with previous reports pointing to FTase as a therapeutic target for treating proteinopathies and suggest that FTIs target dystrophic neurites by enhancing lysosome function and promoting endolysosome trafficking.

We compared brain-penetrant FTIs LNK-754 and lonafarnib because both are known to activate lysosomes and autophagy in mouse models of proteinopathies and have been investigated clinically for cancer treatment [8, 9, 41, 55–57]. Lonafarnib was of particular interest because it markedly reduces tau inclusions [9], is FDA-approved for the treatment of progeria, and has a known safety and adverse event profile in humans [57]. Nonetheless, we found that LNK-754 was considerably more efficacious than lonafarnib towards improving AD pathology of the 5XFAD mouse. Evidence that both LNK-754 and lonafarnib cross the blood brain barrier has come from human clinical cancer trials [41, 57] but a comparison of LNK-754 and lonafarnib blood brain permeability in 5XFAD mice has yet to be performed, so it is conceivable that lonafarnib did not cross the blood brain barrier as efficiently as LNK-754. The discrepancy between LNK-754 and lonafarnib could also be due to differences in potency of LNK-754 and lonafarnib, as the IC50 for H-Ras inhibition is 0.57 nM for LNK-754 [41] and 1.9 nM for lonafarnib [51]. We readily detected changes in known markers of FTase inhibition in the brains of LNK-754 treated 5XFAD mice, but not in lonafarnib mice, supporting the idea that a higher dosage of lonafarnib may be required to achieve a therapeutic effect.

High doses of FTIs are necessary to maximally suppress farnesylation of Ras as well as other canonical CAAX FTase substrate proteins. A low dose (1 mg/kg/day) treatment regimen was followed in this study because evidence indicates that low doses of FTIs can activate non-canonical protein substrates that are involved in protein clearance pathways [8, 28]. Furthermore, low dosage treatment in patients can avoid side effects typically seen with high levels of Ras inhibition. LNK-754 treatment (0.9 mg/kg/day) reduced α-syn in the brains of PD transgenic mice and elevated lysosomal activity above baseline levels in wild-type mice [8]. Conversely, intermittent treatment of lonafarnib by oral gavage at a dose that was substantially higher (80 mg/kg/day) was necessary to inhibit tau aggregation [9]. A higher dose of lonafarnib may be required to enhance both endolysosomal function and axonal trafficking since we found elevated levels of LAMP1 in the brains of lonafarnib treated mice but minimal effects on dystrophies and plaque burden. We tested only a single dosage of FTIs, and further studies will be required to determine the dosage and route of administration required to achieve optimal reduction of Aβ and dystrophic neurite pathologies. The in vitro effects of LNK-754 and lonafarnib were consistent, suggesting that lonafarnib would generate similar phenotypes in 5XFAD mice if comparable inhibitor activity on FTase was achieved. It is possible that drug exposure in cell cultures was more similar for the two compounds, as compared to the in vivo model.

If comparable FTase inhibition occurred in vivo, another possibility is that LNK-754 and lonafarnib altered the farnesylated proteome in the 5XFAD brain in distinct ways. This has been observed in leukemia cell lines, where clear differences in farnesylated proteins were reported following treatment with the FTIs BMS-241,662 and L-778,123 [24]. Additionally, this study demonstrated unique patterns of farnesylation in different cell lines, indicating that a specific farneslyated proteome could exist in each cell type, which could exacerbate small differences in protein farnesylation inhibition between LNK-754 and lonafarnib. Therefore, it is possible that farnesylated proteins specifically inhibited by LNK-754, but not lonafarnib, are responsible for mediating plaque and dystrophy reduction. An alternative possibility, although unlikely [58], is that off-target inhibition of gernyl-gernyl transferase (GGTase) by LNK-754 or lonafarnib could have resulted in differential inhibition of substrates regulated by GGTase. Finally, prenylation pathways for substrates involved in plaque reduction could be activated in lonafarnib-treated mice but not LNK-754 mice. It is likely that the simultaneous inhibition of multiple substrates by LNK-754 led to plaque reduction in 5XFAD mice, and some or all of these substrates were not inhibited in lonafarnib mice. To identify mechanisms of farnesylated substrates in regulating plaque growth and seeding, future experiments should be performed to identify which substrates are most reduced in hippocampal and cortical brain regions, and correlate with decreased plaque burden and dystrophies in the brains of LNK-754 and lonafarnib treated 5XFAD mice.

A trend was observed throughout our chronic study where significant differences were found between vehicle and LNK-754 in the cortex, and between lonafarnib and LNK-754 in the hippocampus. We observed significant decreases in plaques and dystrophies cortical brain regions between vehicle and LNK-754 treated mice, while significant differences between LNK-754 and lonafarnib were found in the hippocampus. Amyloid pathology begins in layer 5 cortical neurons and is most severe in this brain region [36] so we suspect this was due to the low number of plaques in the hippocampus and variability in plaque burden in 5XFAD mice. We expect that our results would reach statistical significance between LNK-754 and vehicle for all outcomes by increasing treatment length or assessing the mice at an advanced age when more plaques would be found in the hippocampus. However, distinct mechanisms may occur between the hippocampus and cortex in LNK-754 treated mice. For example, differences in physiological levels of FTase in cells concentrated in the hippocampus compared to the cortex, or differential expression of FTase substrates may exist between the hippocampus and cortex.

To investigate the mechanism of LNK-754 to reduce amyloid pathology in 5XFAD mice, we focused our study specifically on dystrophic neurites. Dystrophic neurites contain high levels of LAMP1 and BACE1, and significantly contribute to extracellular plaque deposition and axonal dystrophy, resulting in synaptic loss, neurodegeneration, and cognitive deficits [14, 16, 18]. By assessing multiple markers of dystrophic neurites, including LAMP1, BACE1 and LT, we found that equally sized plaques have fewer dystrophies in LNK-754 mice compared to vehicle or lonafarnib treated mice. A moderate reduction in the ratio of LAMP1:Aβ42 per plaque was observed compared to the greater decreases of other dystrophic neurite markers in LNK-754 treated mice, possibly because a portion of plaque-associated LAMP1 is present in microglia [59]. LT accumulated in dystrophies around plaques in living mouse brain tissues (Fig. 4A), showing that highly acidified late endosomes and lysosomes are present in dystrophic neurites. Furthermore, we showed that LNK-754 treatment had a dramatic effect on decreasing the ratio of LT:TR, compared to the ratio of LAMP1:Aβ42. Based on our findings with LT staining, we hypothesize that the subset of LAMP1-positive vesicles that were reduced in LNK-754 chronically treated mice were acidified late endosomes and lysosomes.

Most importantly, LNK-754 prevented the abnormal accumulation of BACE1 in dystrophies. Inhibiting BACE1 activity in dystrophic neurites in the early stages of Aβ deposition suppresses the formation of new plaques, rather than the growth of the original plaque [18], so a similar mechanism could occur in LNK-754 treated mice. In the APP/PS1 mouse model, dystrophic neurites plateau after plaques reach a radius of 10μm, which may be explained by complete axon loss balanced with dystrophic neurite generation [18]. The positive correlations between LAMP1 and plaque burden after chronic LNK-754 treatment, and LT and plaque size after acute LNK-754 treatment, could be caused by a shift in the equilibrium of dystrophy loss and generation, an increase in the number or size of viable axons and neurons surrounding plaques, or a decrease in the abundance of large plaques. A key finding of our study was that LNK-754 and lonafarnib dramatically enhanced retrograde axonal trafficking of endolysosomal compartments. It has been reported that FTIs, including lonafarnib, increase MT acetylation and stability in cancer cell lines [31, 32, 60]. Therefore, the enhanced trafficking of endolysosomal compartments in our study was potentially mediated through substrates responsible for the MT-stabilizing effects of FTIs. Interestingly, a synergistic effect between traditional MT stabilizing agents and FTIs has been well-documented in cancer settings [32, 33, 61], which raises the intriguing possibility that a similar treatment could prevent dystrophic neurite formation in AD brains. The additive effects of enhancing the degradative capacity of lysosomes and trafficking of these compartments likely promoted the targeting of BACE1 to mature lysosomes for degradation.

It is noteworthy that acute treatment with LNK-754 attenuated dystrophic neurites in aged 5XFAD mice and improved spatial learning and memory in hAPP/PS1 mice. This supports a dual role of FTIs in the treatment of AD, by inhibiting Aβ generation and protecting against dystrophic neurites and cognitive decline after advanced plaque deposition. In line with this, haplodeficiency of FTase specifically in APP/PS1 mice rescued cognitive impairment, while deficiency of FTase or GGTase both reduced Aβ deposition [62]. Further, the level of farnesylation has been shown to regulate synaptic plasticity and spine density in hippocampal slices in wild-type mice [63], so farnesylated proteins specifically may alter synaptic plasticity and microtubule stability, and these substrates could function independently of other prenylated substrates that affect plaque deposition. While we hypothesize that farnesylated substrates regulating microtubule stability and endolyosome function improve dystrophic neurites and rescue behavior in the APP/PS1 mouse, other mechanisms are possible. For example, Rhes is a small GTPase that undergoes farnesylation and is known to induce autophagy [64]. Rhes mediated behavior improvement in tau transgenic mice by lowering phospho-tau levels [9], and in a similar manner Rhes could be responsible for improving memory in the LNK-754 treated hAPP/PS1 mouse by reducing tau phosphorylation (e.g., p-tau ser404), as observed in LNK-754 treated 5XFAD mice, independently of dystrophic neurite improvement.

Farneslyation is critical for the proper functioning of multiple physiologically relevant proteins that could be responsible for mediating the beneficial effects observed in our study. Over 32 farnesylated substrates were recently identified through metabolic labeling in a human endothelial cell line [65], and in another recent study, 11 farnesylated proteins were enriched in forebrain-neuron specific FTase knockout mice [63]. A major family of proteins affected in forebrain neurons by farnesylation are Ras GTPases [63], which mediate many cellular functions including endosomal trafficking. Ras proteins are implicated in AD pathogenesis; in postmortem brain tissues of patients with AD and mild cognitive impairment, increased activation of Ras signaling correlates with amyloid pathology and elevated levels of its downstream signaling molecule ERK [30]. Furthermore, the Ras family member Rhes was identified as a target of lonafarnib to activate lysosomes and reduce mutant tau aggregates [9]. Transcriptomics of neuronal FTase knockout in APP/PS1 mice identified mTORC1 signaling and promotion of non-amyloidogenic processing as the top contributor to the beneficial effects on cognition and amyloid pathology, possibly mediated through a reduction in Ras or Rheb signaling [30]. Changes in Rhes or Rheb activity could reduce Aβ burden in 5XFAD mice, although it is unlikely that Ras was completely inactivated at the LNK-754 and lonafarnib concentrations used in this study. Several non-canonical non-Ras FTase substrates could instead mediate the observed effects. For example, the SNARE protein ykt6 enhances the trafficking of lysosomal hydrolases and could promote lysosomal function [8]. A direct FTase effect could also be responsible, since FTase can directly bind microtubules and affect the activity of the histone deacetylase HDAC6, thereby preventing tubulin deacetylation [33].

## Conclusions

Upregulation of FTase signaling and protein farnesylation appears to be an early initiating event in AD pathogenesis [30]. In this study, we take a pharmacological approach to inhibit FTase in vivo in the 5XFAD mouse model. In mouse models of synucleinopathies and tauopathies, toxic protein aggregates were cleared directly by enhanced lysosomal degradation induced by FTI treatment [8, 9]. In contrast, we hypothesize that Aβ generation was inhibited through an indirect mechanism where enhancing axonal trafficking inhibited dystrophic neurite formation and BACE1 elevation. After the onset of plaque pathology, short-term LNK-754 treatment reduced dystrophic neurites in 5XFAD mice, and improved memory and spatial learning behaviors in hAPP/PS1 mice. Our results provide a proof-of-concept study towards the use of FTIs to prevent Aβ accumulation and add to the substantial body of work that has demonstrated FTase genetic knockdown improves amyloid pathology and rescues cognitive function in AD mouse models [30, 62, 66]. The specific FTase substrates and downstream pathways responsible for the phenotypes observed in our study should be further elucidated in future studies. Lowering BACE1 buildup in dystrophic neurites with FTI treatment may slow the deposition of Aβ42 while offering a therapeutic strategy that avoids the off-target side effects typically seen when using BACE1 inhibitors [67, 68]. Furthermore, reducing amyloid plaque burden in the brain will likely predict clinical benefit in AD patients [67], and as such, FTIs could have important therapeutic implications for AD.

## Supplementary Information

**Additional file 1: Supplementary Fig. 1.** Chronic treatment with FTI LNK-754 reduces amyloid burden in the brains of 5XFAD mice. Confocal immunofluorescence microscopy showing uncropped images from Fig. 1A of brain sections from 5-month-old 5XFAD mice treated with vehicle, LNK-754 or lonafarnib immunostained for Aβ42 (red) and NeuN (blue). Scale bar, 1000 μm

**Additional file 2: Supplementary Fig. 2.** Chronic treatment with LNK-754 reduces fibrillar Aβ in the brains of 5XFAD mice. Fibrillar plaque cores were assessed by Thiazine Red immunostaining in the cortex and hippocampus in 5-month-old 5XFAD mice chronically treated with vehicle, LNK-754 or lonafarnib. Quantifications are shown of plaque size in the hippocampus (*p = 0.022 between vehicle and LNK-754, ***p = 0.0003 between LNK-754 and lonafarnib) **(A)** and cortex **(B)** and of plaque count in the hippocampus **(C)** and in the cortex (*p = 0.049) **(D)**. Vehicle, *n* = 11 (5 males, 6 females); LNK-754, *n* = 10 (4 males, 6 females); lonafarnib *n* = 10 (4 males, 6 females). Triangles represent males and circles represent females. 1-way ANOVA with Tukey’s post-hoc multiple comparisons test was performed.

**Additional file 3: Supplementary Fig. 3.** LNK-754 reduces amyloid burden and tau pathology in female and male 5XFAD mice. **A** Immunoblot of brain homogenates from vehicle, LNK-754 and lonafarnib treated 5XFAD mice probed for APP (6E10) and actin. Quantification of APP **(B)** in male **(C)** and female **(D)** mice. **E** Immunoblot of brain homogenates from male vehicle, LNK-754 and lonafarnib treated 5XFAD mice probed for phospho-tau (P-tau ser404) and total tau (Tau1). Quantification of phospho-tau (P-tau ser404) normalized to total tau in male and female (immunoblots shown in Fig. 1H) mice (*p = 0.012) **(F)**. Vehicle, *n* = 11 (5 males, 6 females); LNK-754, *n* = 10 (4 males, 6 females); lonafarnib *n* = 10 (4 males, 6 females). Triangles represent males and circles represent females. 1-way ANOVA with Tukey’s post-hoc multiple comparisons test and 2-way ANOVA were performed.

**Additional file 4: Supplementary Fig. 4.** Effect of LNK-754 and lonafarnib on brain levels of markers of farnesyltransferase inhibition. Immunoblot of brain homogenates from vehicle, LNK-754 and lonafarnib treated 5XFAD mice probed for prelamin-A **(A),** HDJ-2 **(B),** phospho-ERK1 (p-P44/P42) and total-P44/P42 (t-P44/P42) **(C)** and actin. Arrow denotes slower migrating HDJ-2 species in **(B)**. Quantifications of prelamin-A **(D)** immunoblots in **(A),** HDJ-2 (*p = 0.036 for vehicle versus LNK-754, *p = 0.023 for LNK-754 versus lonafarnib) **(E)** immunoblots in **(B)** (upper band), p-P44/P42 **(F)** immunoblots in **(C)**. Signals were normalized to actin and expressed as fold of vehicle. Triangles represent males and circles represent females. Vehicle, *n* = 11 (5 males, 6 females); LNK-754, *n* = 10 (4 males, 6 females); lonafarnib *n* = 10 (4 males, 6 females). 1-way ANOVA with Tukey’s post-hoc multiple comparisons test was performed.

**Additional file 5: Supplementary Fig. 5.** LNK-754 and lonafarnib increase total LAMP1 levels in the brains of 5XFAD mice. **A** Immunoblot of brain homogenates from vehicle, LNK-754 and lonafarnib treated 5XFAD mice probed for LAMP1 and actin. Quantifications of LAMP1 **(B)** immunoblots in **(A).** Signals were normalized to actin and expressed as fold of vehicle (***p = 0.0009 between vehicle and LNK-754, ****p < 0.0001 between vehicle and lonafarnib)**.** Quantification of total LAMP1 fluorescence intensity levels, shown in Fig. 2A and B, in the cortex (*p = 0.029 between vehicle and LNK-754, *p = 0.026 between vehicle and lonafarnib) **(C)** and hippocampus (**p = 0.0054 between vehicle and LNK-754, *p = 0.011 between vehicle and lonafarnib) **(D)** of vehicle, LNK-754 and lonafarnib treated 5XFAD mice. Quantification of dystrophic neurite LAMP1 fluorescence intensity levels, shown in Fig. 2A and B, in the cortex **(E)** and hippocampus **(F)** of vehicle, LNK-754 and lonafarnib treated 5XFAD mice. Vehicle, *n* = 11 (5 males, 6 females); LNK-754, *n* = 10 (4 males, 6 females); lonafarnib *n* = 10 (4 males, 6 females). Triangles represent males and circles represent females. 1-way ANOVA with Tukey’s post-hoc multiple comparisons test was performed.

**Additional file 6: Supplementary Movie 1.** LysoTracker-Green trafficking in vehicle-treated wild-type neurons.

**Additional file 7: Supplementary Movie 2.** LysoTracker-Green trafficking in LNK-754-treated wild-type neurons.

**Additional file 8: Supplementary Movie 3.** LysoTracker-Green trafficking in lonafarnib-treated wild-type neurons.

## Data Availability

The datasets used and/or analysed during the current study are available from the corresponding author on reasonable request.

## Abbreviations

BACE1: β-site amyloid precursor protein cleaving enzyme
FTIs: Farnesyltransferase inhibitors
AD: Alzheimer’s disease
Aβ: Amyloid-β
FTase: Farnesyltransferase
α-synuclein: Alpha-synuclein
PD: Parkinson’s disease
MT: Microtubule
i.p: Intraperitoneal
LAMP1: Lysosomal-associated membrane protein 1
LT: LysoTracker-Green
MWM: Morris Water Maze
