# Supplementary figures and images for: Farnesyltransferase inhibitor LNK-754 attenuates axonal dystrophy and reduces amyloid pathology in mice
Source: Mol Neurodegener. 2022 Aug 20;17:54. doi: 10.1186/s13024-022-00561-9 (PMC9392365; doi:10.1186/s13024-022-00561-9)

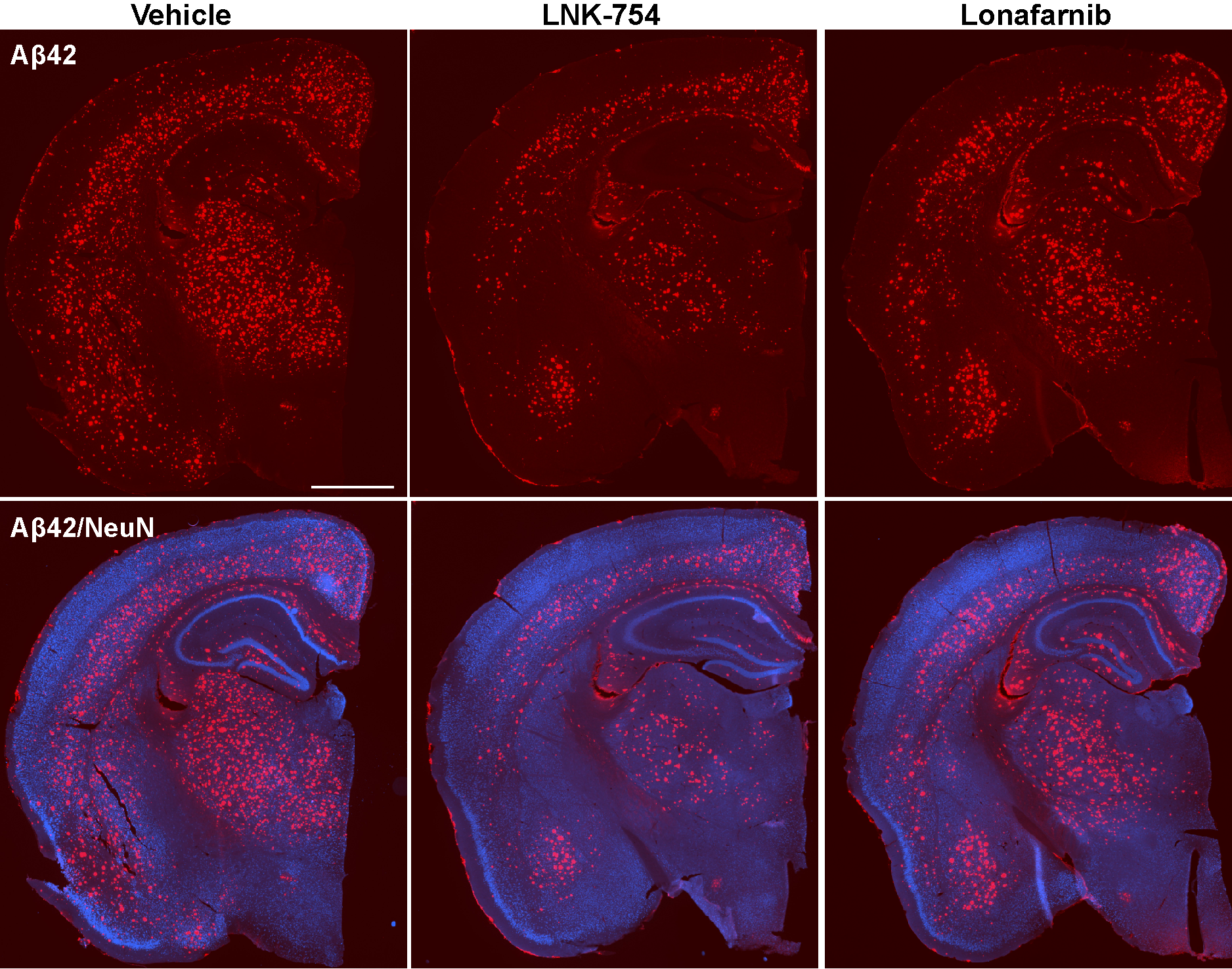

Supplement: Supplementary file 1 — Additional file 1: Supplementary Fig. 1. Chronic treatment with FTI LNK-754 reduces amyloid burden in the brains of 5XFAD mice. Confocal immunofluorescence microscopy showing uncropped images from Fig. 1A of brain sections from 5-month-old 5XFAD mice treated with vehicle, LNK-754 or lonafarnib immunostained for Aβ42 (red) and NeuN (blue). Scale bar, 1000 μm [file 13024_2022_561_MOESM1_ESM.tif]

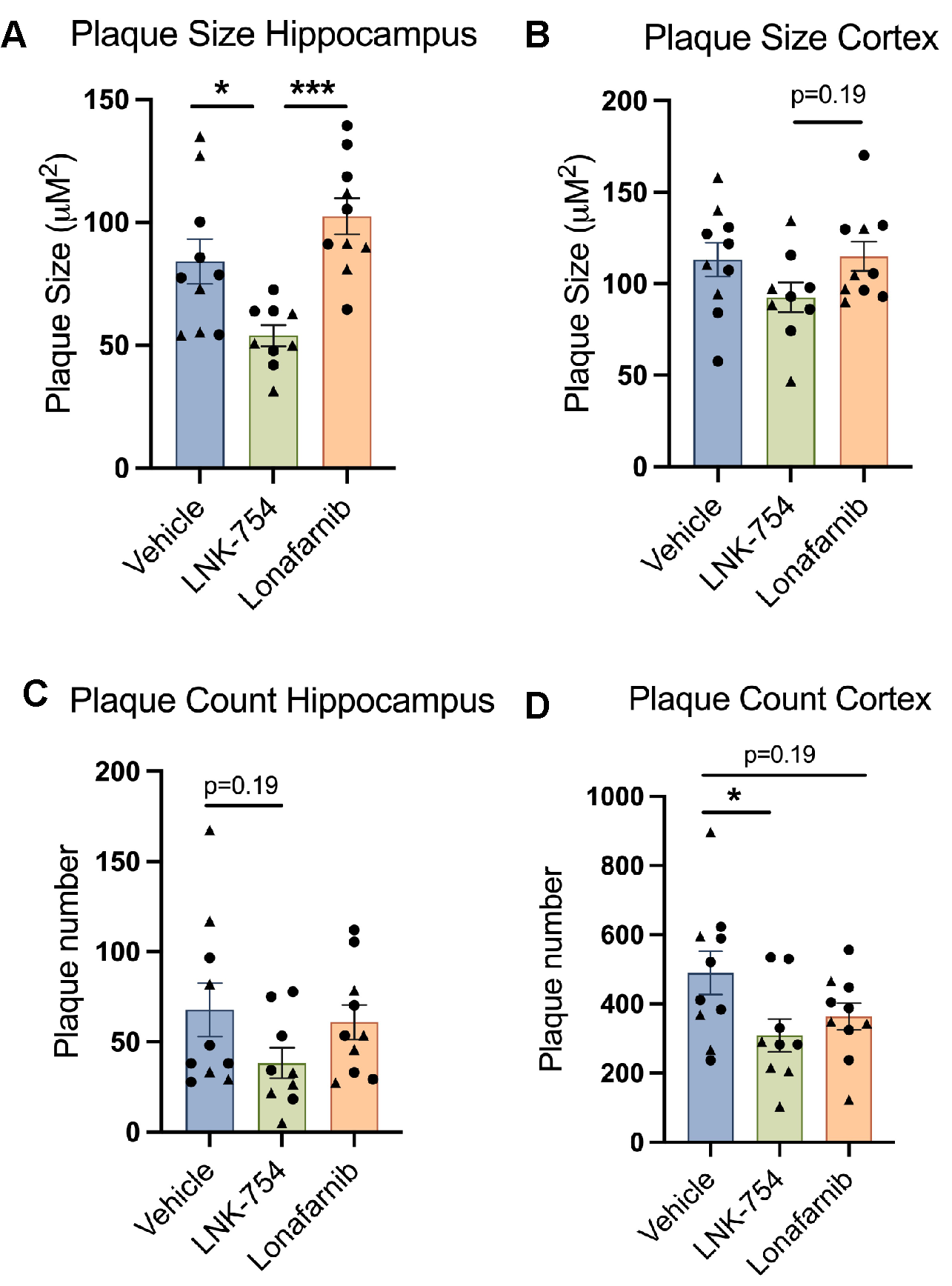

Supplement: Supplementary file 2 — Additional file 2: Supplementary Fig. 2. Chronic treatment with LNK-754 reduces fibrillar Aβ in the brains of 5XFAD mice. Fibrillar plaque cores were assessed by Thiazine Red immunostaining in the cortex and hippocampus in 5-month-old 5XFAD mice chronically treated with vehicle, LNK-754 or lonafarnib. Quantifications are shown of plaque size in the hippocampus (*p = 0.022 between vehicle and LNK-754, ***p = 0.0003 between LNK-754 and lonafarnib) (A) and cortex (B) and of plaque count in the hippocampus (C) and in the cortex (*p = 0.049) (D). Vehicle, n = 11 (5 males, 6 females); LNK-754, n = 10 (4 males, 6 females); lonafarnib n = 10 (4 males, 6 females). Triangles represent males and circles represent females. 1-way ANOVA with Tukey’s post-hoc multiple comparisons test was performed. [file 13024_2022_561_MOESM2_ESM.tif]

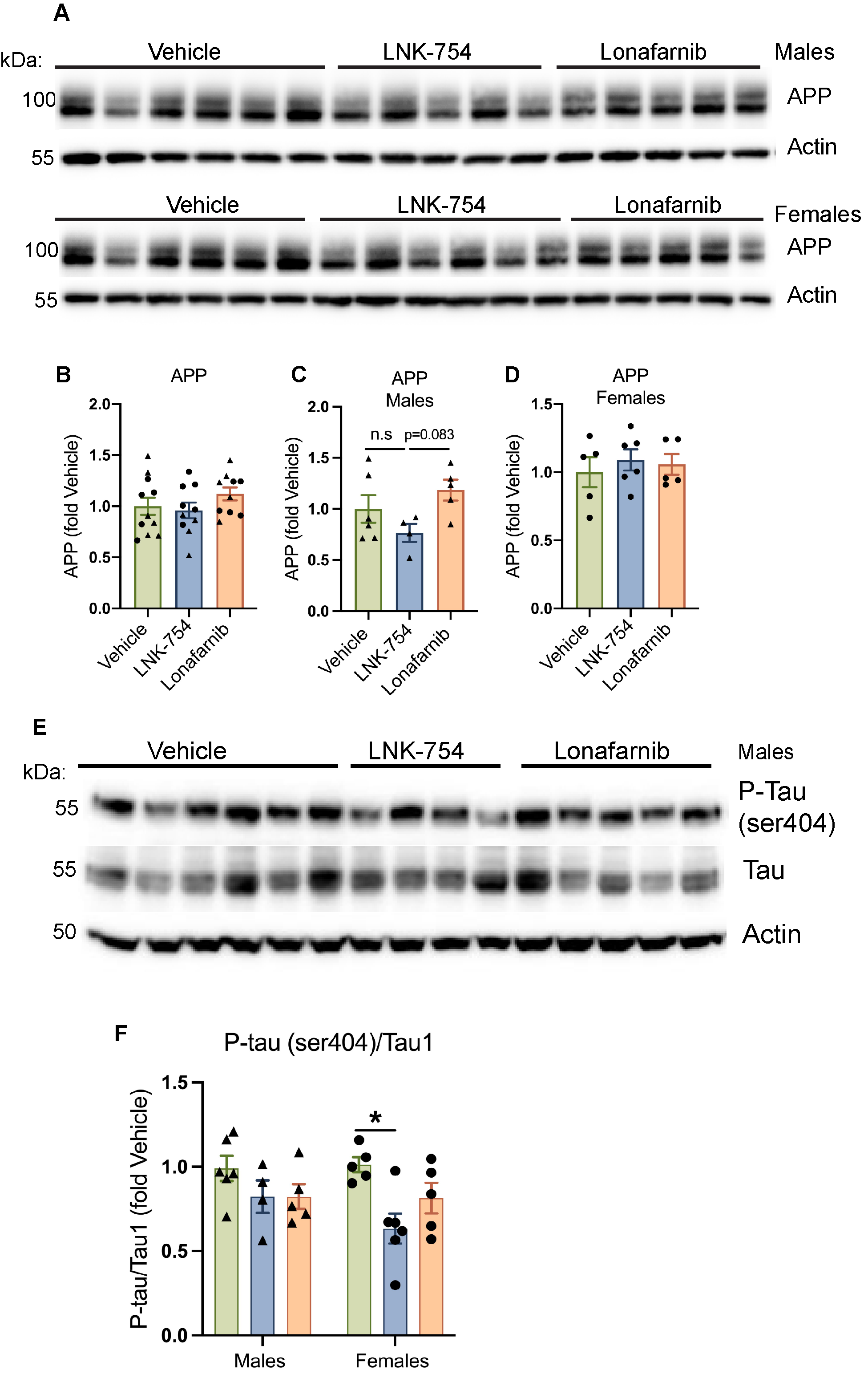

Supplement: Supplementary file 3 — Additional file 3: Supplementary Fig. 3. LNK-754 reduces amyloid burden and tau pathology in female and male 5XFAD mice. A Immunoblot of brain homogenates from vehicle, LNK-754 and lonafarnib treated 5XFAD mice probed for APP (6E10) and actin. Quantification of APP (B) in male (C) and female (D) mice. E Immunoblot of brain homogenates from male vehicle, LNK-754 and lonafarnib treated 5XFAD mice probed for phospho-tau (P-tau ser404) and total tau (Tau1). Quantification of phospho-tau (P-tau ser404) normalized to total tau in male and female (immunoblots shown in Fig. 1H) mice (*p = 0.012) (F). Vehicle, n = 11 (5 males, 6 females); LNK-754, n = 10 (4 males, 6 females); lonafarnib n = 10 (4 males, 6 females). Triangles represent males and circles represent females. 1-way ANOVA with Tukey’s post-hoc multiple comparisons test and 2-way ANOVA were performed. [file 13024_2022_561_MOESM3_ESM.tif]

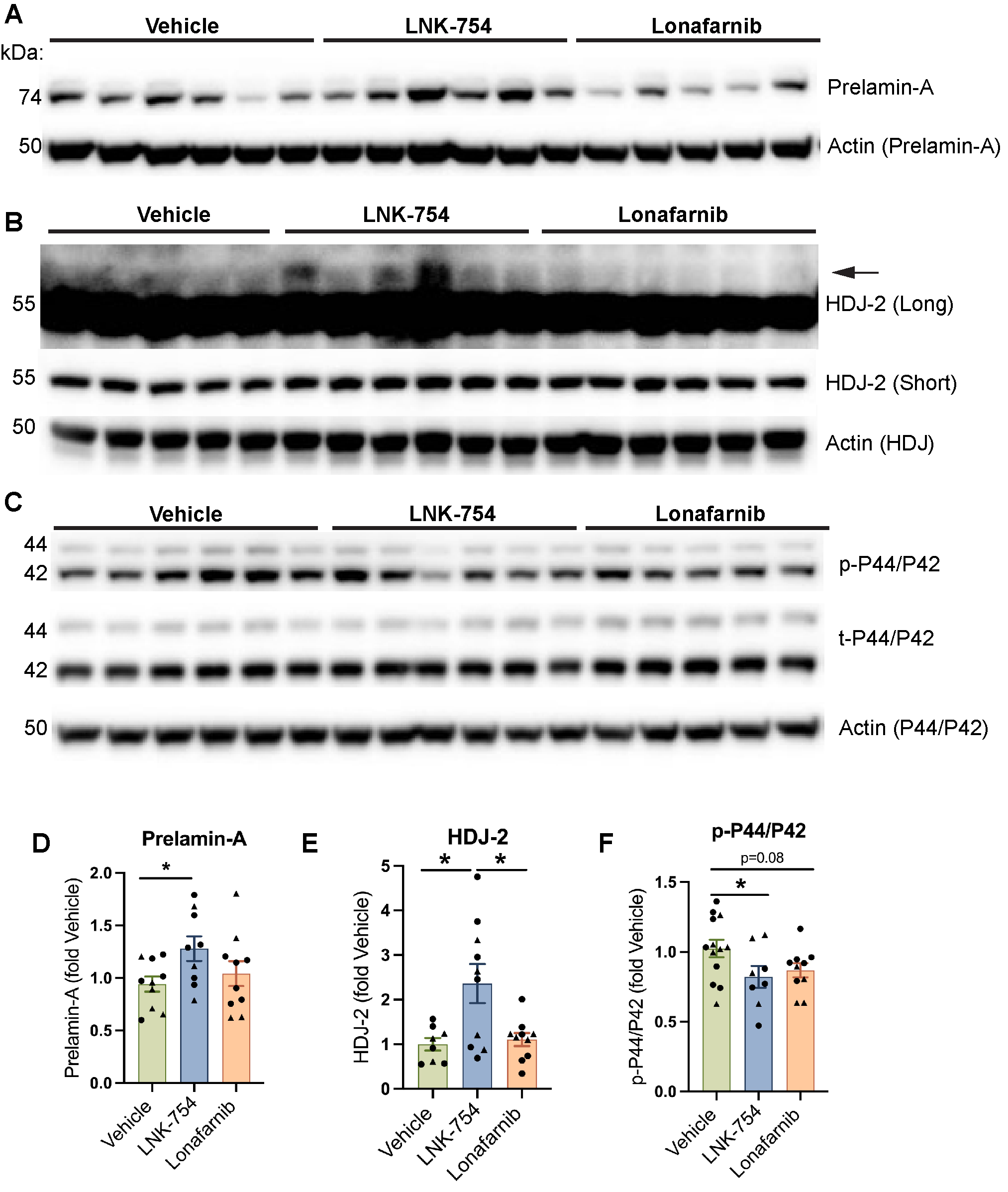

Supplement: Supplementary file 4 — Additional file 4: Supplementary Fig. 4. Effect of LNK-754 and lonafarnib on brain levels of markers of farnesyltransferase inhibition. Immunoblot of brain homogenates from vehicle, LNK-754 and lonafarnib treated 5XFAD mice probed for prelamin-A (A), HDJ-2 (B), phospho-ERK1 (p-P44/P42) and total-P44/P42 (t-P44/P42) (C) and actin. Arrow denotes slower migrating HDJ-2 species in (B). Quantifications of prelamin-A (D) immunoblots in (A), HDJ-2 (*p = 0.036 for vehicle versus LNK-754, *p = 0.023 for LNK-754 versus lonafarnib) (E) immunoblots in (B) (upper band), p-P44/P42 (F) immunoblots in (C). Signals were normalized to actin and expressed as fold of vehicle. Triangles represent males and circles represent females. Vehicle, n = 11 (5 males, 6 females); LNK-754, n = 10 (4 males, 6 females); lonafarnib n = 10 (4 males, 6 females). 1-way ANOVA with Tukey’s post-hoc multiple comparisons test was performed. [file 13024_2022_561_MOESM4_ESM.tif]

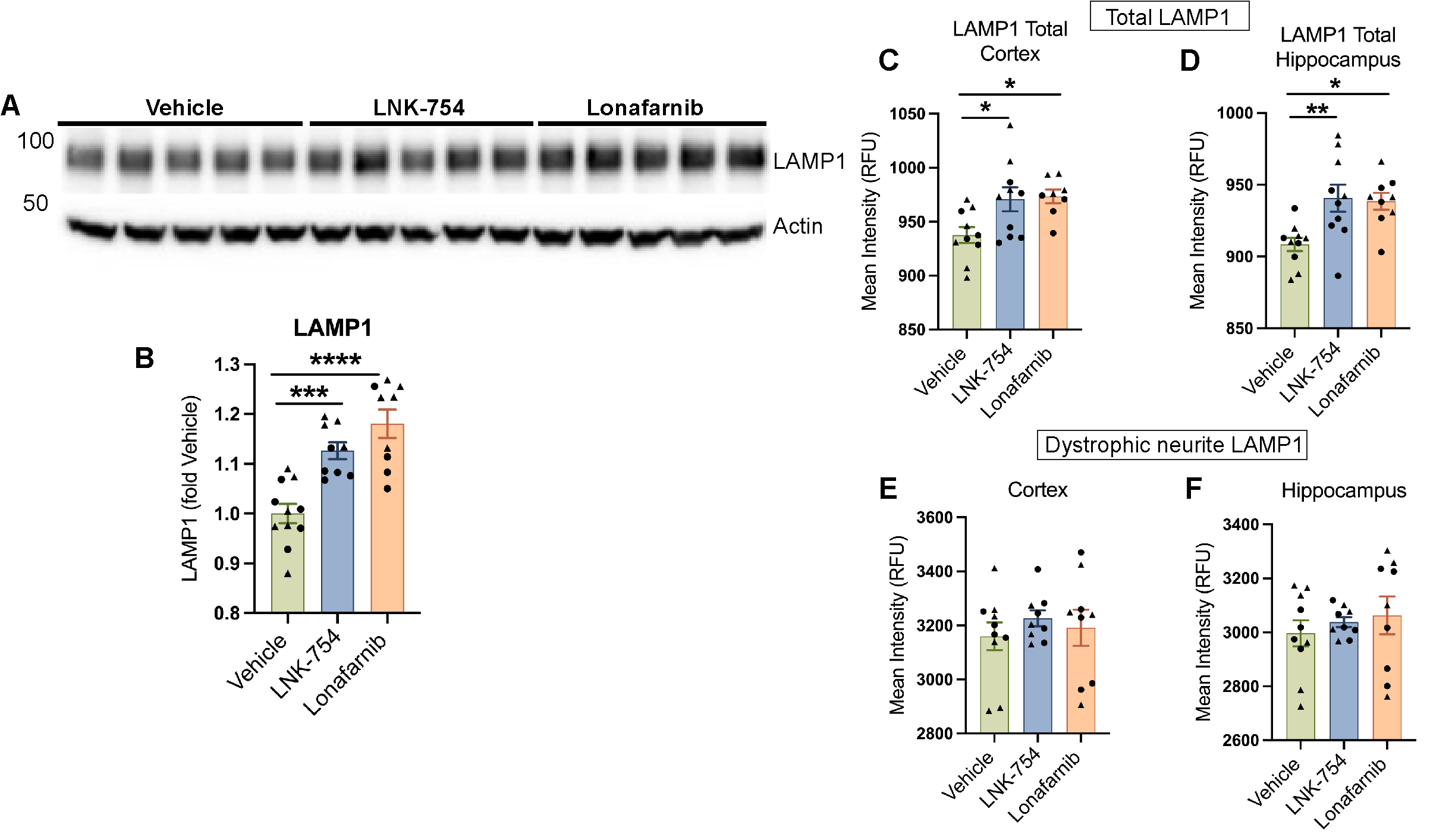

Supplement: Supplementary file 5 — Additional file 5: Supplementary Fig. 5. LNK-754 and lonafarnib increase total LAMP1 levels in the brains of 5XFAD mice. A Immunoblot of brain homogenates from vehicle, LNK-754 and lonafarnib treated 5XFAD mice probed for LAMP1 and actin. Quantifications of LAMP1 (B) immunoblots in (A). Signals were normalized to actin and expressed as fold of vehicle (***p = 0.0009 between vehicle and LNK-754, ****p < 0.0001 between vehicle and lonafarnib). Quantification of total LAMP1 fluorescence intensity levels, shown in Fig. 2A and B, in the cortex (*p = 0.029 between vehicle and LNK-754, *p = 0.026 between vehicle and lonafarnib) (C) and hippocampus (**p = 0.0054 between vehicle and LNK-754, *p = 0.011 between vehicle and lonafarnib) (D) of vehicle, LNK-754 and lonafarnib treated 5XFAD mice. Quantification of dystrophic neurite LAMP1 fluorescence intensity levels, shown in Fig. 2A and B, in the cortex (E) and hippocampus (F) of vehicle, LNK-754 and lonafarnib treated 5XFAD mice. Vehicle, n = 11 (5 males, 6 females); LNK-754, n = 10 (4 males, 6 females); lonafarnib n = 10 (4 males, 6 females). Triangles represent males and circles represent females. 1-way ANOVA with Tukey’s post-hoc multiple comparisons test was performed. [file 13024_2022_561_MOESM5_ESM.tif]
